# Two Indias: The structure of primary health care markets in rural Indian villages with implications for policy

**DOI:** 10.1016/j.socscimed.2020.112799

**Published:** 2022-05

**Authors:** Jishnu Das, Benjamin Daniels, Monisha Ashok, Eun-Young Shim, Karthik Muralidharan

**Affiliations:** a Georgetown University, Washington, DC, USA; b Center for Innovation and Impact, Global Health Bureau, USAID, USA; c Amazon, USA; d University of California, San Diego, USA; e Centre for Policy Research, New Delhi, India

**Keywords:** India, Quality of care, Human resources for health, Informal, Providers, Health markets

## Abstract

We visited 1519 villages across 19 Indian states in 2009 to (a) count all health care providers and (b) elicit their quality as measured through tests of medical knowledge. We document three main findings. First, 75% of villages have at least one health care provider and 64% of care is sought in villages with 3 or more providers. Most providers are in the private sector (86%) and, within the private sector, the majority are ‘informal providers' without any formal medical training. Our estimates suggest that such informal providers account for 68% of the total provider population in rural India. Second, there is considerable variation in quality across states and formal qualifications are a poor predictor of quality. For instance, the medical knowledge of informal providers in Tamil Nadu and Karnataka is higher than that of fully trained doctors in Bihar and Uttar Pradesh. Surprisingly, the share of informal providers does not decline with socioeconomic status. Instead, their quality, along with the quality of doctors in the private and public sector, increases sharply. Third, India is divided into two nations not just by quality of health care providers, but also by costs: Better performing states provide higher quality at lower per-visit costs, suggesting that they are on a different production possibility frontier. These patterns are consistent with significant variation across states in the availability and quality of medical education. Our results highlight the complex structure of health care markets, the large share of private informal providers, and the substantial variation in the quality and cost of care across and within markets in rural India. Measuring and accounting for this complexity is essential for health care policy in India.

## Highlights

• In 2010, the average Indian village had 3.2 primary healthcare providers.
• Of these, 86% were in the private sector and 68% had no formal medical training.
• In richer states, the share of informal providers did not decrease—quality improved.
• In high-performing states, quality was higher *and* per-patient costs were lower.
• The density and variation of health market cost and quality has policy implications.

## 1 Introduction

One view of primary care in rural India is that it is available mainly through publicly-operated Primary Health Care Centers or Sub-Centers, which are sparsely located and under-staffed. According to this view, qualified doctors in India are mostly located in urban locations and access to quality care in rural India is poor (Anand and Fan, 2016; Nandan and Agarwal, 2012; Rao et al., 2011). An alternate view agrees that even though access to *qualified* providers in rural India is low, a wide variety of health care providers with diverse qualifications has arisen to fill this gap (De Costa and Diwan, 2007; Kanjilal et al., 2007). According to this view, primary health care in rural India is delivered through a vibrant, dense, and competitive marketplace that includes few qualified providers with an MBBS degree (the equivalent of an MD in the United States), but a wide variety of non-MBBS providers including AYUSH providers (alternative medical practitioners with degrees in Ayurveda, Yoga, Unani, Siddhi and Homeopathy) as well as those with other or no medical qualifications. *Average* quality in this marketplace may be low, but considerable variation in quality implies that the rural population still has multiple choices among low- and high-quality providers located in (or close to) every village.

Depending on their beliefs about the availability of treatment options in rural India, the quality of public and private sector providers, and the link between medical qualifications and quality, states in India are adopting a wide variety of policies to improve health care. States like West Bengal and Chhattisgarh are trying to improve quality by including those without formal training within the ambit of the public system: either by training informal providers (West Bengal) or by creating an alternate cadre of rural health care providers with a reduced 3-year training requirement (Chhattisgarh) (Downie, 2017; Raha et al., 2010). States like Uttar Pradesh and Madhya Pradesh are trying to improve access to high-quality care in hospitals by providing transportation links that decrease the cost of access, or by focusing on district hospitals (Babiarz et al., 2016; DailyRounds, 2015). At the national level, the Government of India's emphasis on “Wellness Centers” attempts to alleviate the shortage of qualified public sector providers in rural India by allowing providers trained in alternative forms of medicine to also provide care in public health care centers (Government of India, 2017). Many of these policies are actively resisted from organizations like the Indian Medical Association, which maintain an unyielding stance regarding the norms of training and health care provision. In their view, for instance, training informal sector providers is “like teaching burglars to steal better” and their influence led to the termination of the Chhattisgarh initiative (Pulla, 2015).

Part of these intellectual disagreements and subsequent variation in policy approaches reflects a simple but troubling fact: We simply do not know what the landscape of rural health care providers really looks like. There is little information on the types of providers practicing in rural India, their qualifications or experience, or the differences in their distribution across villages and states. There is limited evidence on what doctors know and how they practice, and the little evidence that we have typically comes from small studies in select states (Das and Hammer, 2005; Gautham et al., 2014; Rao et al., 2013). We have virtually no data on fees and patient loads in the private sector, where 75% of Indians seek primary care, and sparse high-quality data on per-visit costs and patient loads in the public sector (Mackintosh et al., 2016). The lack of data on these fundamental aspects of the health system implies that highly consequential policy decisions around health care provision in rural India are based on a limited picture of what is truly happening on the ground.

To address this gap, we provide the first nationwide picture of the availability and quality of primary health care in rural India. We do so in three parts. First, using surveys we conducted in 1519 Indian villages between 2008 and 2010 for the 19 most populous Indian states (except Delhi), we provide estimates of the availability of health care providers in an average village disaggregated by qualifications and sector (public or private). We then augment the nationwide data on provider availability with additional information on providers’ knowledge of how to diagnose and treat four different health conditions that we collected using medical vignettes: tuberculosis in a young adult, preeclampsia in a pregnant woman, diarrhea in a young child and dysentery in a young child. Medical vignettes for these conditions, among others, have been psychometrically validated and used in several previous studies in India, but their use has been restricted to small samples in a handful of states. Our survey presents the first nationwide picture of provider knowledge with key information on how this knowledge differs across states and medical qualifications. Finally, we also collect data on provider caseload and fees (in the private sector) as well as staff salaries (in the public sector). These data allow us to compute the unit cost of each patient visit, allowing us to examine both quality and cost in a unified framework.

We report three main sets of results. First, most Indian villages have access to a health care provider in the village: 74% of villages in our sample had at least one health care provider and 64% of care is sought in villages with 3 or more providers. Most providers (86%) are in the private sector. In terms of qualifications, of the 3473 providers who were surveyed, 2367 (68%) were informal providers in the private sector (IPs), 842 were AYUSH providers (24%) and 264 (8%) had an MBBS degree. We demonstrate the surprising result that this share of informal providers does not decline significantly with measures of state-level development as measured by an index of socioeconomic status.

Second, we find striking variation in provider knowledge, our main measure of provider quality, across Indian states. Average provider knowledge in Tamil Nadu and Kerala is over two standard deviations higher than in Uttar Pradesh and Bihar. This is akin to moving a provider from the average to the top (97th percentile) of the knowledge distribution. Further, even though the stated aim of medical education is to increase medical knowledge, qualifications are only weakly correlated with medical knowledge. We find that fully qualified MBBS doctors from states like Uttar Pradesh and Bihar have lower levels of knowledge than unqualified providers in states like Tamil Nadu and Kerala. An important related finding is that there is strong positive correlation across the knowledge of all types of providers within a given geographical area. The greater availability of knowledgeable public sector doctors is not associated with a reduction in the prevalence of informal unqualified providers, but rather an increase in their medical knowledge. Consequently, at higher levels of socioeconomic status the share of informal providers does not decline – their knowledge improves. This strong co-movement of quality in the public and private sector is a major finding in this paper that helps explain the ubiquity of the informal sector – but it could only be uncovered once we directly measured quality of all types of providers in rural India.

Third, while the fact that levels of provider knowledge are higher in Tamil Nadu and Kerala may not be that surprising, what is surprising is that the better performing states also deliver this quality at a lower cost per patient. In other words, it is not just that public expenditure on health is higher in these states, but that the expenditure is more efficient. One reason is less unused capacity among health care providers due to higher utilization in these states; an intriguing possibility is that utilization itself is higher because quality is also higher. Our results thus show that India is divided into two countries in terms of availability and quality of care, but not in the way that is usually imagined. The “Two Indias” are, in fact, on different *production frontiers* – Northern states are stuck in a situation with low quality and high per-visit costs while Southern states enjoy higher quality at *lower* per-visit costs.

One interesting implication of the “Two Indias” result is that the same policies can lead to different impacts in different states. We illustrate this with two simple thought experiments, in which we (a) incorporate AYUSH providers into the public sector and (b) increase the availability of public primary care. In the better performing states, these policies have little effect on quality or cost because the public and private systems are highly comparable along both dimensions. In the poorer performing states, the effects are large and unpredictable; in some cases, they increase costs substantially without any effect on quality and in others they have a quality payoff without a substantial impact on costs. We hypothesize that investments in medical education may have contributed to increasing the overall supply and quality of health care providers in the better performing states, thereby reducing costs of provision of health care in the state, regardless of whether the provider is in the public or private sector.

Our research contributes both to our understanding of the availability of primary health care in India as well as the quality of primary care providers. Previous attempts to quantify the availability of human resources in India have followed one of two approaches. One approach that allows for national and sub-national estimates uses census classification of occupations at the district level with ancillary evidence from employment categories in India's National Sample Survey (Anand and Fan, 2016; Rao et al., 2011). A second set of studies relies on dedicated surveys of health care providers with a focus on a restricted geographical area such as a district or an urban neighborhood.^1^ Unfortunately, the census and the dedicated survey approach can lead to very different estimates of the overall availability and types of providers, even in the same area. This reflects, in part, that census occupational categories are not designed to capture the variety of health care providers in India; “allopathic doctors”, for instance, include both fully trained medical professionals *and* providers without any formal training.^2^ But it also reflects differences in counts arising from other, unknown reasons. For the state of Madhya Pradesh a dedicated survey counted twice as many providers as the census estimates, suggesting substantial biases that alter our understanding of rural health care availability in India.^3^

Unlike human resource availability where we have at least some estimates, there are no nationwide data on quality. Starting from 2007, researchers have sought to measure quality in select samples using specialized tools. In Delhi, Das and Hammer (2005) first measured the medical knowledge of health care providers in the public and private sector and complemented these measurements with direct clinical observations in 2008 (Das and Hammer, 2005). The method of medical vignettes that they developed has since been used in samples from other Indian states, including Chhattisgarh, Madhya Pradesh, Andhra Pradesh, Uttar Pradesh and Bihar (Das and Hammer, 2005; Gautham et al., 2014; Mohanan et al., 2015; Rao et al., 2013). In 2016 Das et al. used the technique of standardized patients to address several shortcomings of quality measurement from direct clinical observations, and since then this technique has also been used – again in select samples from Madhya Pradesh, Bihar, Maharashtra, West Bengal and Delhi (Das et al., 2016a; Das et al., 2016b; Das et al., 2015; Kwan et al., 2018; Mohanan et al., 2015). These measurements have helped inform discussions around quality of care in India, but there has been no attempt to compare quality across states (these studies were not sufficiently coordinated to permit such comparisons), with the absence of quality measurement from states in South India a particularly important omission from these studies.

Our combination of a dedicated, village-level health care provider survey with a focus on national representativeness can therefore augment our understanding of the availability and distribution of health care providers at local levels across the country. The patterns we uncover confirm the findings from small-sample provider studies both in terms of the unavailability of qualified providers and the ubiquity of informal providers, but differences across states are surprising and different from what is usually imagined. One key result is that an established North-South divide in administration, availability of medical colleges and child mortality is also reflected in quality and costs. We hypothesize why this is so and show that this divide has fundamental implications for how policies will play out in these states.

The remainder of the paper is as follows. Section 2 presents the context of health care in India and Section 3 introduces the data and methods. Section 4 discusses the findings regarding the availability of primary care, the knowledge of primary care providers and results from our policy simulations. Sections 5, 6 present the limitations and conclusions.

## 2 Context

India's post-independence health care system can be traced back to Joseph P. Bhore's committee report in 1946. The report envisioned a U.K.-style, tax-funded National Health System with care delivered through salaried doctors in a three-tiered public system consisting of Primary Health Clinics (PHCs) in villages, Community Health Centers (CHCs) for more complex cases, and district and city hospitals for tertiary care (Patel et al., 2011).

The committee further envisioned that all providers would be trained in allopathic medicine over a period of 5.5 years (including 1 year of internship), after which they would be granted an MBBS degree, which is equivalent to an MD in the U.S. This vision of standardized and uniformly high quality care did not create a space for licentiate medical practitioners with a shorter training duration, who were the main providers of rural care at the time of independence. Neither did it allow for AYUSH providers, who are trained in Indian systems of health care such as Ayurveda or Unani. The implementation of the report recommendations and oversight of the health care system was left to the states with a limited role for the federal government. See Rao et al. for an overview of these historical developments (Rao et al., 2011).

Over the next 70 years, three critical tensions in the systems became apparent. First, it became clear that the country could not produce the number of MBBS doctors that would be required to provide adequate care to the entire country. In an ongoing process, the government “regularized” physicians who were trained in Indian medical systems, eventually allowing colleges to be set up for their training, and granting them recognition as AYUSH providers with a dedicated Ministry of AYUSH established in 2014.

Second, a limited federal role combined with significant geographical disparities posed a challenge to a nationalized and standardized health care service. Take medical training: the Medical Council of India (MCI) is the sole body governing medical education in all Indian states and it sets minimum standards for medical colleges in terms of the infrastructure, faculty, curricula and evaluation criteria. However, individual colleges are responsible for designing and administering MBBS exams and there is no centralized exit examination that imposes a uniform criteria on the knowledge of graduating MBBS students (Medical Council of India, n.d.a, n.d.b, Medical Council of India, n.d.c, Medical Council of India, n.d.d).

By 2016, there were 412 medical colleges with a total capacity of 51,690 undergraduates and 28,111 post-graduates, half of them set up after 2001 (Ravi et al., 2017). Despite the rapid expansion, relative capacity remains much lower than in the U.S., which has a quarter of India's population: the US medical school system produces nearly 20,000 new graduates *each year* from 151 medical schools with a total enrollment around 80,000 (Association of American Medical Colleges, 2019). The spatial distribution of medical colleges remains highly skewed, with 152 in the six South Indian states and only 43 in the Eastern states of Bihar, Jharkhand, Orissa and West Bengal, even though the two regions each account for around 20% of total population. Finally, there have been many concerns among medical professionals and in the media about the quality of education in medical colleges, in part due to an acute shortage of teachers and clinical materials for teaching purposes. Medical colleges are subject to MCI inspection, but there is a widespread perception that these inspections can be gamed, for instance, by recruiting faculty members and patients only during the inspection period (Ananthakrishnan, 2010, 2007; Macaskill et al., 2015; Naik, 2014).

Third, the continued shortage of MBBS doctors combined with regional disparities led to a medical landscape where the public sector never emerged as a dominant source of care. In 2014, the private sector accounted for 75% of all primary care visits and the private share of total health expenditures was 70% in 2012. Out-of-pocket health expenditures, which amounted to 7% of household expenditures in 2012, contributed 61% of total health expenditures (Karan et al., 2014; Mackintosh et al., 2016). Medical care in the private sector – and increasingly the public sector – is provided by a variety of providers, ranging from fully trained MBBS doctors to AYUSH and informal providers, the latter without any formal training. Governments are increasingly turning to non-MBBS doctors to satisfy demand; for instance, 501 AYUSH educational institutions with an additional capacity of 26,790 students had been established by 2010 and AYUSH facilities have been co-located with district hospitals, CHCs, and PHCs in most states, intertwining the quality of AYUSH education with the provision of care in the public sector (Government of India, 2010). Further, regional disparities in health outcomes and in the provision of primary care have persisted. Child mortality of 7 per 1000 in Kerala is comparable to OECD countries, while child mortality of 58 per 1000 in Bihar and 78 per 1000 in Uttar Pradesh are similar to some of the poorest countries in Sub-Saharan Africa (International Institute for Population Sciences, 2016). To what extent these regional disparities reflect the availability and quality of medical care is currently unknown.

India's size and heterogeneity thus provides a fertile environment to examine key questions of how health systems, seen through the lens of its human resources, varies across states within a single federation. With the data that we have collected, we are able to both describe the existing health environment at a level of detail that has not been attempted previously and examine critical questions regarding health systems development.

## 3 Data and methods

### 3.1 Sampling

The Medical Advice Quality and Availability in Rural India (MAQARI) study was conducted in the 19 most populous states in India, excluding Delhi, between 2008 and 2010. The sample is representative of more than 90% of India's rural population. Within each state, 10 districts were selected using probability proportional to size (PPS) based on population and within each sampled district, 8 villages were selected. The eventual sample covered 1519 villages.^4^

To ensure broad geographical coverage, each state was divided into distinct socio-cultural regions (SCRs); states have between 3 and 8 SCRs depending on their size. Once the state was divided into SCRs, the 10 districts were allocated across the SCRs proportional to population so that larger SCRs had more sampled districts. The stratification ensured that the study was representative across all major SCRs in the country. Once the number of districts was assigned to each SCR, districts were selected within the region using PPS. Since the study is on health care in rural India, districts with more than 60% urban population were removed from the sampling frame.

All sampling was conducted on the basis of the 1991 census and all analysis was weighted by the inverse of sampling probability, so the final estimates continue to be nationally-representative on a population weighted basis. Within each state, the sampling probability of villages were proportional to their population, thus, the sampling weights assigned to villages were needed only for aggregation across states. We adjust for the state rural population size by assigning a weight to each village, denoted by $W_{village}$ and calculated as:

$$W_{village}=\frac{SCR\;rural\;population}{Number\;of\;villages\;in\;the\;SCR}$$

Details about probability weights used in the analysis of the paper can be found in Appendix A.

### 3.2 Measurement methods

To assess the availability of care in rural Indian villages, a village-mapping approach was used. Between October 2009 and December 2009, enumerators visited each of the 1519 sampled villages and mapped all the primary care providers, regardless of their sector (public or private) or qualifications (MBBS, AYUSH or unqualified).^5^ For every provider in this final list, we completed a questionnaire with information on medical training, experience, and details of the provider's medical practice. A total of 8942 providers were included in the census of village providers. After excluding chemists/pharmacists, dental care providers, and Anganwadi and Accredited Social Health Activist (ASHA) workers (whose main function is public health education and maternal health) we were left with 4335 providers. Appendix Table A1 shows that survey completion among these 4335 enumerated providers was 80%, with non-completion attributable mainly to the provider being away rather than due to refusals. As temporary absences from clinics are a frequent occurrence in rural India, we interpret the final sample as showing the availability on an average day or week in these villages (Chaudhury et al., 2006).

In addition, we also completed a questionnaire among randomly sampled households in the village, which we use to compute an index of socioeconomic status (SES) for each state. This SES index was computed based on household caste, school attainment of adult household members, residence characteristics such as roofing type, electricity connection, and access to piped water, and ownership of mobile phone, electric fan, pressure cooker, bicycle, and television. Household-level variables were transformed to a one-dimensional index by principal components analysis (Filmer and Pritchett, 1998). Appendix Table A2 provides summary statistics of the household characteristics used in the calculation of the SES index.

Finally, medical knowledge was measured using clinical vignettes with each provider completing three of four conditions – tuberculosis in a young adult male, preeclampsia in a pregnant mother and either diarrhea or dysentery in a young child (half the providers received the dysentery case and the other half diarrhea). These cases had been developed and tailored to the Indian context previously, with agreed-upon definitions of what constitutes a necessary checklist of history questions and examinations and correct case management for each case (Das and Hammer, 2005; Das et al., 2015). Following Das and Hammer (2005), we base our measure of knowledge on each provider's adherence to the case-specific checklist of necessary history questions and examinations. Each necessary history or examination item is appropriately weighted for difficulty using item response theory and the estimated knowledge scale is then standardized to have a mean of 0 and a standard deviation of 1. In the results, we first benchmark our continuous measure of knowledge against specific tasks and overall diagnosis and treatment for each of the vignettes cases. We use a lenient definition of correct management, which includes referral to higher levels of care, but does not penalize the use of unnecessary medicines or even antibiotics.

In addition to the providers drawn from the village-based samples, a separate part of the vignettes study assessed provider knowledge in a representative sample of Primary and Community Health Care centers (PHC/CHC) in the same districts. To retain the focus on rural health care, we do not generally use the results from this sample. The only exceptions are when we construct district or state-level measures of knowledge for MBBS providers. In this case, the small number of rural MBBS providers renders the village-based sample imprecise (in half of the states, we found less than 10 MBBS providers in all villages combined) and we therefore also include the sample of MBBS providers tested at PHCs and CHCs. Because this sample is only used to present aggregated state-level means for MBBS providers, we defer the sampling discussion to the Supplemental Appendix. In the two cases that we use this sample, we clearly indicate both in the text and the exhibit notes that we have done so, referring to this as the “PHC/CHC vignettes sample”.

### 3.3 Measurement challenges

The biggest challenge, and one we discuss further in Section 5.2 below, is the construction of markets. Our village-level estimates of availability and quality are accurate as long as people seek primary care only within the village. Our research in Madhya Pradesh (Das and Mohpal, 2016) showed that 34% of care was sought outside villages and that there is a trade-off between distance travelled and provider knowledge so that visits outside the village were to more knowledgeable providers. To the extent that *urban* areas, which we did not sample, represent a small fraction of primary care visits, our estimates remain unbiased both for the availability of health care within the village *and* the average quality of health care for patients in rural areas in each state. But they are likely underestimates of the availability and quality of health care in every village. We discuss in Section 5.2 that, in the absence of administrative data, there is no clear solution for how to construct primary care health markets and we believe that this is one of the major challenges for research on rural health care moving forward.

Further, the sample for providers with medical vignettes differs from the census of provider availability for two reasons. First, for budgetary reasons, we covered a sample rather than census of providers in each village. Specifically, a maximum of six providers per village were sampled from the provider listing. If the village had six or fewer providers, all providers were sampled. If the village had more than six providers, one public provider was randomly sampled and the remaining five were sampled from the pool of both public and private providers. Because there were fewer public providers, this sampling procedure ensured that at least one public provider was included in the sample if there was one in the village.

Second, we were unable to complete the medical vignettes with a significant fraction of sampled providers. This non-completion is mainly a result of our two-stage survey strategy, where in the first stage we enumerated providers in each village using a short questionnaire and in the second stage, we sampled from this list and returned some months later to implement the clinical vignettes. We had successfully implemented this strategy in smaller studies, but it turned out to be problematic in a large nationwide study (J. Das and Hammer, 2007b; Das and Mohpal, 2016). Health care providers are often difficult to identify (*“The provider whose clinic is by the big tree in the market”*); they are frequently absent or travelling; and people use different identifiers for the same provider. Our budget was insufficient to maintain a dedicated field team for each state and therefore when the team returned, they were able to definitively locate and complete 59.5% of providers who had been selected for the vignettes. We discuss potential limitations for interpretation in Section 5 and analyze missingness further in Appendix C.

For the vignettes that were completed, we found the data to be of uniformly high quality and in the absence of any other comparable nationwide data, results based on this exercise provide a starting point for key discussions. We acknowledge, however, that even though our results are robust to traditional parametric techniques that account for non-completion (such as inverse-probability weights), non-parametric methods such as bounding exercises are uninformative with this high rate of non-completion. We therefore advocate caution in (over)interpreting these results; for completeness we present the sampling procedure, the sample and completion proportions in the Supplemental Appendix.

## 4 Findings

We first present four key findings related to the availability, qualifications, workload and state-level variation in the availability of human resources for primary care in India's villages. We then present data on the distribution of knowledge across states. Finally, we examine three policy proposals regarding primary care in the country.

### 4.1 The availability and per-visit cost of primary rural health care providers

**Fig. 1:**
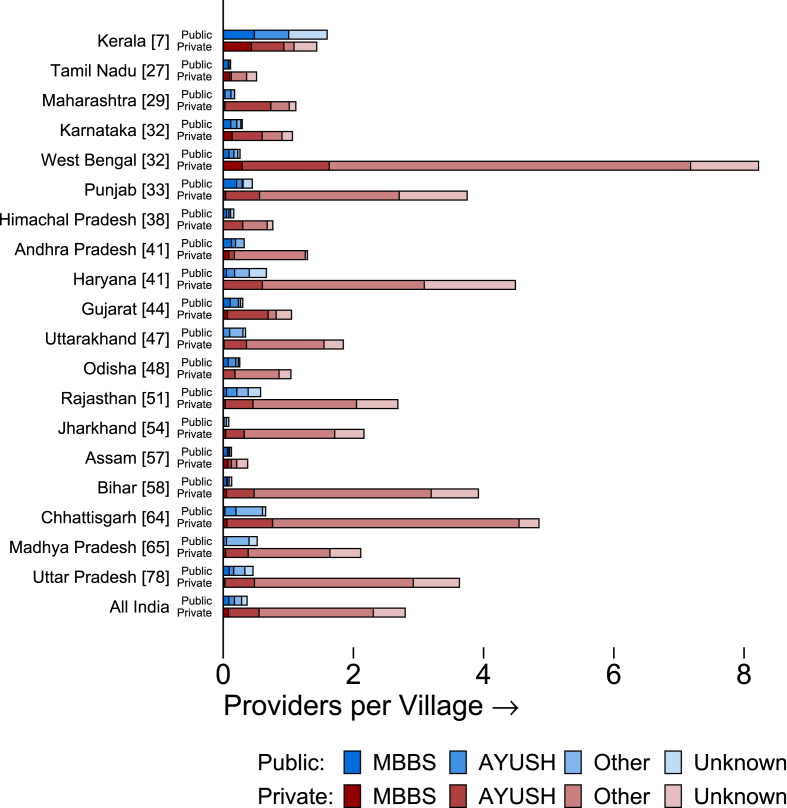
Average availability of providers at the village level. Notes: This figure reports the number of health care providers of each type and sector available in the average village in each state. States are ordered by the under-five mortality rate per 1000 live births over the five-year period covered by the National Family Health Survey (NFHS-4) for 2015–16 (reported in brackets). Health care providers are defined as surveyed workers at health care facilities, excluding chemists and paramedical staff such as nurses/GNMs, ANM/VHNs, MPW/MNAs, and compounders and assistants. They are categorized by the sector (public or private) and by the type of formal medical education reported: an MBBS degree, or the equivalent of an MD; an AYUSH degree (a licensed practitioner of alternative or traditional medicine falling under Ayurveda, Yoga, Unani, Siddha, or Homeopathic systems); other, or no recognized formal medical degree; and unknown, or no response. *Data source:* village provider census.

Fig. 1 and Table 1 report village-level provider availability for each state using the appropriate sampling weights. In Fig. 1, states are sorted in the order of under-five mortality rates (U5MR).^6^ We definitively located 4335 providers in the sampled villages, or an average of 3.2 primary care providers per village. Of these, 3739 were in the private sector (86%) and the remaining 596 in the public sector (14%). We were then able to survey and identify qualifications for 3473, as the remainder were unavailable. Fig. 1 reports the corresponding distribution of qualifications for each state and for the full Indian sample, where providers who were unavailable are reported as “unknown”. Of the 3473 providers who were surveyed, 2367 (68%) were informal providers in the private sector (IPs), 842 were AYUSH providers (24%) and 264 (8%) had an MBBS degree. For context, the average village in our sample has 2670 inhabitants, 75% of villages have fewer than 3000, and 90% of villages have under 6000 residents.

**Table 1:** Average availability of providers at the village level. Notes: This table reports the proportion of villages in each state in which at least one health care provider was identified in various categories, and the proportion of villages in which no health care providers were observed. N = 4335 health care providers, observed across 80 villages per state, except Uttar Pradesh (88), Punjab (79), and Uttarakhand (72). *Data source:* village provider census.

	(1)	(2)	(3)	(4)	(5)
	Public MBBS	Private MBBS	Any MBBS	Any Non-MBBS	No Providers
Andhra Pradesh	11.6%	7.6%	15.7%	61.8%	34.6%
Assam	3.8%	7.4%	11.2%	51.2%	47.6%
Bihar	3.9%	3.8%	7.6%	96.2%	3.8%
Chhattisgarh	2.6%	4.1%	6.8%	91.2%	7.5%
Gujarat	9.5%	4.1%	11.2%	44.3%	55.7%
Haryana	5.0%	0.0%	5.0%	96.2%	3.8%
Himachal Pradesh	5.0%	0.0%	5.0%	41.3%	56.3%
Jharkhand	0.0%	3.7%	3.7%	72.4%	26.4%
Karnataka	11.5%	7.0%	18.6%	47.0%	48.5%
Kerala	37.9%	29.0%	56.1%	87.2%	7.0%
Madhya Pradesh	0.8%	3.8%	4.5%	88.9%	11.1%
Maharashtra	2.6%	2.6%	4.0%	61.5%	38.5%
Odisha	4.3%	0.0%	4.3%	72.2%	27.8%
Punjab	16.5%	3.8%	17.8%	96.1%	3.9%
Rajasthan	5.0%	3.3%	7.2%	81.7%	18.3%
Tamil Nadu	6.6%	7.6%	11.9%	38.7%	57.7%
Uttar Pradesh	3.7%	2.7%	5.2%	96.1%	2.3%
Uttarakhand	0.0%	1.3%	1.3%	68.4%	31.6%
West Bengal	6.3%	19.3%	22.9%	97.4%	2.6%
India Total	**6.4%**	**6.0%**	**10.9%**	**75.0%**	**23.4%**

Table 1 shows what the distribution of providers by qualification implies for the average village in each state. Across all states, in our sample of villages, 6.4% report access to a public and 6.0% report access to a private MBBS provider in that village. Since some villages have both, across all 1519 villages, only 10.9% have access to any MBBS provider, whether public or private. In sharp contrast, 75% of villages have access to an informal provider (IP) in the village, and we additionally compute that 64% of primary care visits occur in villages with 3 or more health care providers.^7^ The majority of patients in rural India can thus access care from multiple sources in competitive markets, even though this care is provided mostly through a network of providers without formal qualifications.

Surprisingly, state-level variation in the number of providers appears to be uncorrelated with health indicators such as child mortality. For instance, Bihar, a state with poor human development outcomes and high U5MR, has 4.0 primary care providers in the average village and a U5MR of 58 per 1000. By contrast, Tamil Nadu has 0.6 providers available in the average village, but has one of the lowest child mortality rates in the country at 7 per 1000. In many states with poor health outcomes (such as Bihar, Chhattisgarh and Uttar Pradesh) fewer than 10% of villages reported no access to a provider within the village, while in states with better health outcomes (Tamil Nadu, Andhra Pradesh, Karnataka) more than a third of villages have no providers at all. As we argue below, the number of providers in a village does not imply anything about their quality: Measures of availability without measures of quality do not correlate with health outcomes.

**Fig. 2:**
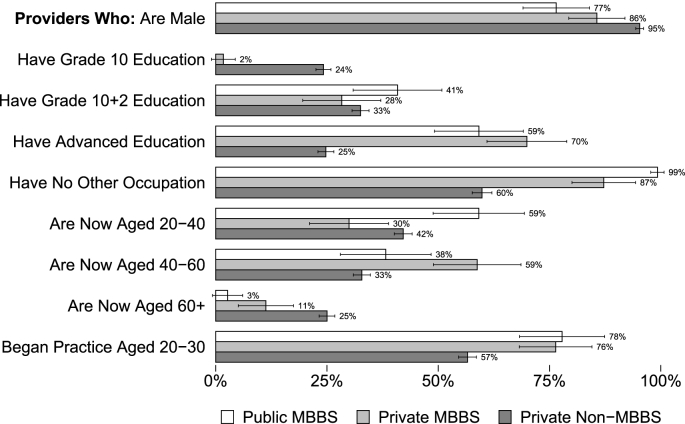
Demographics of village health care providers. Notes: This figure reports the share of health care providers in each of three groups: Public sector providers who hold an MBBS degree (N = 139); private sector providers who reported holding an MBBS degree (N = 125); and all other private providers including those reporting AYUSH degrees, those reporting other or no degree, and unknown (N = 2923). *Data source:* village provider census and survey.

Fig. 2 shows that the health care providers surveyed, regardless of degree or sector, were overwhelmingly male (from 77% of public providers to 95% of private non-qualified providers). A majority reported no other occupation (60% of private non-qualified providers to 99% of public providers). Of particular interest is that most providers without an MBBS degree reported secondary or higher education (24% reported less than secondary education), confirming that education cannot be used as a proxy for qualifications in census estimates. Furthermore, the majority started practice between the ages of 20 and 30 with little difference by qualification. Since the average age is close to 40, half of all providers, including IPs, have been practicing for at least 10 years. Substantial tenure implies that providers view their jobs as permanent and are not operating as fly-by-night operators who enter and exit the market rapidly. IPs appear to have a permanent presence in the villages that they practice in.

**Fig. 3:**
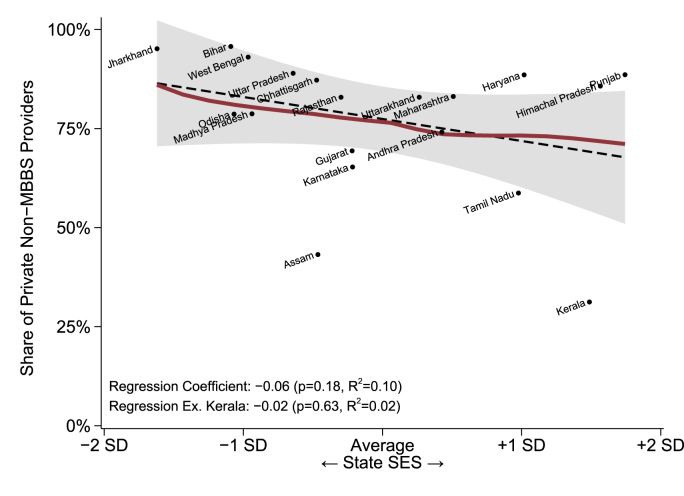
Socioeconomic status and non-MBBS prevalence. Notes: This figure plots the average share of private non-MBBS providers in each state against the standardized average socioeconomic status of households in the state calculated from the household wealth survey. The dotted black line reports the full-sample (N = 19) relationship between average SES and the share of non-MBBS providers with 95% confidence intervals; the solid line reports a nonparametric (LOWESS) fit. Univariate regression results are reported including and excluding the observation for Kerala. The share of non-MBBS providers is calculated as the number of private sector providers who reported AYUSH degrees or those who reported other or no degrees, as a share of the total number of public and private sector providers who reported their degree (excluding providers whose qualifications were unknown). *Data source:* village provider census and survey; household survey; village vignettes sample.

Fig. 3 shows that the dominance of non-MBBS providers (AYUSH and IPs) declines very slowly with the level of economic development, measured by the socioeconomic status (SES) of our household sample in each state. Non-MBBS providers are a minority in only two states – Assam and Kerala. In contrast, they make up more than half of all providers in the remaining states, ranging from states like Bihar and Uttar Pradesh, where health outcomes are extremely poor, to states like Punjab and Himachal Pradesh, where health outcomes are notably better. While higher state SES predicts a smaller presence for non-MBBS providers, the association is weak and driven primarily by the inclusion of Kerala. Moving from the lowest to the highest value of state SES is associated with a reduction from 86% to 68% in the share of non-MBBS private providers, or 5.6p.p. per SES standard deviation (*p* = 0.18). Excluding Kerala from this regression reduces the association further to 1.8p.p. per SES standard deviation – the predicted shares then range from 83% to 77% with state SES. Any regression model implies a large majority share for non-MBBS providers at all SES levels and, in general, the variation across states at any given SES level is greater than the variation by SES.

Despite the uniform presence of informal providers across states, per-visit costs differ substantially. To compute an aggregate per-visit cost, we first use self-reported fees in the private sector, weighted by the private share of visits in the state. We then add the per-visit cost in the public sector, again weighted by the visit share in the public sector to the private sector fees. Since providers in the public sector are paid a monthly salary, the per-visit cost in the public sector is computed as the ratio of total compensation to patients seen. Consequently, it differs both due to variation in compensation and variation in the number of patients: A PHC with one doctor and a salary of Rs.20,000 will have a per-visit cost of Rs.200 if he sees 100 patients a month, but only Rs.20 if he sees 1000. Note that relative to the private sector, where fees incorporate all costs to the provider, in the public sector we exclude infrastructure and administrative costs, thus providing a lower bound of the cost per patient.

Table A4 shows this variation, both in compensation and patient load across states. In Gujarat, the average monthly salary of an MBBS doctor in the public sector in our survey is Rs.91,000 but the monthly patient load is only 489. In contrast, the monthly compensation of an MBBS doctor in Kerala in the public sector is Rs.15,919 with a monthly patient load of 1292. Combined with a high share of public sector visits, this makes Kerala one of the lowest cost states in our sample with a per-visit cost of Rs.9 and Gujarat one of the highest cost with a per-visit cost of Rs.97.

The most surprising component of our cost computations is the finding of wide variation in reported patient loads across states, which is at odds with a perception that rural health care providers are over-burdened. In fact, our data show that most rural providers operate at substantial excess capacity. Based on self-reports, the average doctor sees 13 patients per day and spends 12 minutes with each, implying that a rural provider spends, on average, two and a half hours a day seeing patients.^8^

**Fig. 4:**
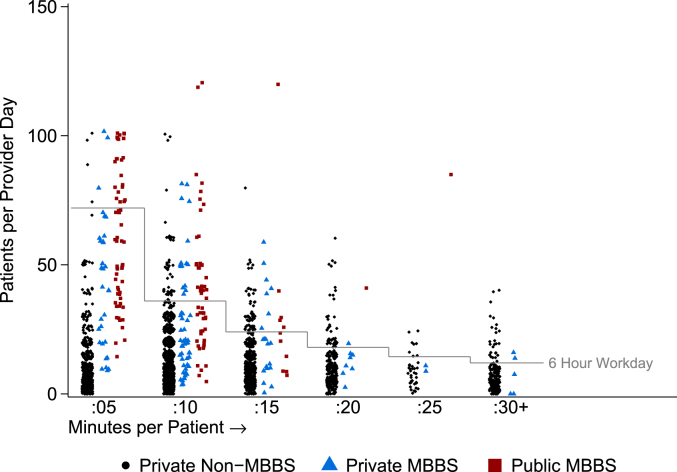
Provider patient load and time utilization. Notes: This figure plots the self-reported number of patients seen per day (continuous) against the self-reported number of minutes spent with each patient (categorical). Each reporting provider is represented by a single marker. The reference line indicates the number of patients that would be required in each duration bin for the provider to spend 6 h per day seeing patients. This figure reports statistics for providers in each of three groups: Public sector providers who hold an MBBS degree (N = 130); private sector providers who reported holding an MBBS degree (N = 122); and all other private providers including those reporting AYUSH degrees, those reporting other or no degree, and unknown (N = 2902). Providers were excluded if they did not report the number of patients (707 providers); if they reported zero time per patient (3 providers); or if they reported more than 120 patients per day (14 providers). Public sector number of patients per day was calculated as the average number of patients per day reported by all providers in a clinic, divided by the number of providers in the clinic. *Data source:* village provider census and survey.

Fig. 4 explores this phenomenon of excess capacity further. We plot the total number of patients seen per day (vertical axis) against the average consultation length (horizontal axis) for every provider in our sample. Movements along the vertical axis at the same consultation length therefore reflect a higher overall workload, and movements along the horizontal axis represent higher consultation lengths. If providers were working at full capacity, we would expect to see a cluster of dots along the reference line (a generous 6 h seeing patients per day). Instead, 8.3% of doctors report workdays longer than 6 h and 4.5% longer than 8 h; the average provider uses 40% of their available time to see patients based on a 6-h workday and 30% based on an 8-h workday. Multiple regression estimates (Table 2) with and without state-level controls and fixed-effects show that public providers, those with an MBBS degree, and those who report no other occupation all report larger patient loads (the latter is most likely endogenously determined by the caseload itself). However, even among public MBBS providers, self-reported hours with patients exceeds 6 h per day for just 38% of providers.

**Table 2:** Regression of correlates of provider caseload. Notes: This table reports regression estimates for the self-reported number of patients seen daily by each provider surveyed. Standard errors are in italics below point estimates. The first column reports the base specification with all included covariates. The second column reports a specification controlling for the state mean of the socioeconomic index. The third column includes a set of fixed effects (indicator variables) with one for each state. Public sector number of patients per day was calculated as the average number of patients per day reported by all providers in a clinic divided by the number of providers in the clinic. *Data source:* village provider census and survey; household survey.

	(1)	(2)	(3)
	Base Regression	State Characteristics	State Fixed Effects
Public Sector	22.51*** 5.05	22.54*** 4.99	22.85*** 4.90
MBBS Degree	11.49*** 2.96	10.19*** 3.02	8.15*** 2.86
Village population size (x100s)	0.11*** 0.00	0.09*** 0.00	0.07*** 0.00
Provider Who: Is Male	0.98 *1.43*	2.52* 1.39	3.14** 1.43
Have Grade 10 Education	−1.09 *2.98*	−1.17 *3.12*	−0.98 *3.32*
Have Grade 10 + 2 Education	1.38 *2.99*	1.32 *3.13*	1.36 *3.32*
Have Advanced Education	2.38 *3.01*	2.52 *3.16*	2.81 *3.34*
Have No Other Occupation	3.81*** 0.48	3.24*** 0.49	2.88*** 0.51
Are Now Aged 40-60	0.94 *0.65*	0.91 *0.64*	0.65 *0.63*
Are Now Aged 60+	−0.99 *1.18*	−0.85 *1.16*	−1.21 *1.16*
State mean of household SES index		5.53*** 0.91	
State Under-5 Mortality Rate		−0.05*** 0.02	
Constant	3.39 *3.26*	−0.13 *3.65*	3.37 *3.57*
Number of Observations	3143	3143	3143
Caseload Mean	14.26	14.26	14.26
Caseload Standard Deviation	17.90	17.90	17.90

Even this is an upper bound of the time that rural health care providers actually spend seeing patients. In the absence of time and motion studies we have had to rely on self-reported data, which exaggerate both patient load and time spent with patients. Indeed, in studies where we have both self-reports and clinical observations, the two are strongly correlated but time-use and patient loads are significantly higher in self-reports compared to direct observations. Appendix Figure A4, for instance, plots self-reports against observed patient loads in a study from West Bengal, clearly demonstrating the extent of over-reporting for providers who self-report seeing more than 5 patients a day. Appendix Figures A5 and A6 suggest that time per patient is also exaggerated in self-reports, part of which may be due to the severe heaping we see at 5, 10, 15, and 25 min.

Our results thus far highlight the high availability of primary care in rural India combined with substantial excess capacity, which in the public sector increases the average cost of each visit. Most primary care is provided by informal providers – private sector providers without MBBS degrees. The share of IPs remains stable across states at very different levels of SES and child mortality. At the same time, visit costs vary widely due to variation in private sector fees, public sector compensation and public sector patient loads. The flat IP share is particularly surprising as most observers argue that the share of IPs will decline as SES increases. However, this declining share will be true only if: (a) SES is associated with the demand for higher quality *and* (b) IPs provide universally low quality care. We next provide evidence that the second part of the argument is what fails in India – rather than their fraction declining, the quality of IPs improves dramatically with state SES.

### 4.2 The knowledge level of health care providers in rural India

Key to the resolution of the puzzle of high IP presence in every Indian state regardless of the level of socioeconomic development is the measurement of provider knowledge. The fundamental insight that emerges is that, across India, providers with the same qualifications can have vastly different medical knowledge. In fact, as state SES increases, so does the medical knowledge of all health care providers.

To recap, our measure of provider knowledge is a weighted index of adherence to a checklist of necessary history questions and examinations for each of 4 cases, of which 3 were presented to each provider in the sample. Here, the weights are derived from an optimal scoring algorithm based on item response theory as in Das and Hammer (2005). The measure is standardized so that the mean provider in the sample has a score of 0 and the distribution has a standard deviation of 1.

In order to understand what this measure implies in terms of concrete tasks, Table A3 benchmarks the knowledge score against essential tasks and correct management for each of the cases. A provider in the third quintile (just above the average in the sample, with a score of +0.13) knows to order a sputum test for a patient presenting with TB in 74.2% of cases and correctly manages TB in 82.7% of cases. This is similar to the proportion who correctly manage the diarrhea/dysentery case by providing ORS (85.0%) but significantly higher than the correct management proportion of 67.1% for preeclampsia. There are meaningful and large differences across quintiles of the knowledge score. Moving from the bottom quintile with a mean knowledge score of −1.61 to the top quintile with a mean knowledge score of 1.33 is associated with a 30–40 percentage point increase in the likelihood of adhering to critical history questions and exams for TB and diarrhea and 60–70 percentage points for preeclampsia. It is also associated with a 30–40 percentage point increase in the likelihood of correctly managing each case.

Table 3 then illustrates the variation in knowledge across states and qualifications. We present multiple regression estimates from various specifications. Column 1 includes provider characteristics – qualifications, sex, age, whether the provider is in the public sector – and the mean SES of households in the district. Column 2 restricts the regression to public providers and Column 3 to private providers only. Column 4 then includes the mean knowledge of *public* providers in the district, where we use the PHC/CHC vignettes sample to construct this additional variable. Column 5 further restricts the sample to IPs only. Columns 6–10 then repeat these specifications with state fixed effects.

**Table 3:** Regression of correlates of provider competence. Notes: This table reports regression estimates for the medical knowledge of each provider surveyed who later participated in the medical vignettes exercise. Standard errors are in italics below point estimates. The first column reports the base specification with all included covariates. The second column reports a specification limited to public providers only. The third and fourth columns report a specification limited to private providers only, with and without an included covariate for the mean competence of public providers in the same district. The fifth column reports a specification limited to private non-MBBS providers with an included covariate for the mean competence of public providers in the same district. Columns 6–10 repeat these specifications and include a set of state fixed effects (indicator variables). The provider competence variable is constructed using IRT on the full sample such that the mean score corresponds to a value of zero and the standard deviation in that distribution is 1. *Data source:* village provider census and survey; household survey; village and PHC/CHC vignettes sample.

	No State Fixed Effects					State Fixed Effects				
	(1)	(2)	(3)	(4)	(5)	(6)	(7)	(8)	(9)	(10)
	Full Sample	Public Providers	Private Providers	Private Providers	Private non-MBBS	Full Sample	Public Providers	Private Providers	Private Providers	Private non-MBBS
**MBBS Degree**	0.56***	0.66***	0.48**	0.33**		0.25*	0.58**	0.07	0.08	
	*0.15*	*0.22*	*0.19*	*0.16*		*0.14*	*0.23*	*0.17*	*0.17*	
**Provider Is Male**	−0.31**	0.19	−0.54***	−0.28***	−0.31***	−0.02	0.36**	−0.23**	−0.19*	−0.23**
	*0.13*	*0.24*	*0.14*	*0.11*	*0.11*	*0.11*	*0.17*	*0.11*	*0.10*	*0*.*10*
**Provider Age**	0.00	−0.02	0.00	0.00	0.00	0.00	−0.03*	0.00	0.00	−0.01
	*0.00*	*0.02*	*0.00*	*0.00*	*0.00*	*0.00*	0.01	*0.00*	*0.00*	*0.00*
**Public Sector**	0.33*					0.07				
	*0.17*					*0.14*				
**Mean District Public Competence**				0.89***	0.88***				0.55***	0.54***
				*0.06*	*0.06*				*0.07*	*0.07*
**District SES**	0.64***	0.15	0.68***	0.06	0.04	0.25	−0.29	0.34*	0.35*	0.31
	*0.09*	*0.23*	*0.10*	*0.10*	*0.10*	*0.19*	*0.46*	*0.20*	*0.20*	*0.20*
**Constant**	−0.64	0.78	−0.52	0.16	0.23	−0.30	1.48	−0.28	−0.19	−0.08
	*0.26*	*0.99*	*0.26*	*0.22*	*0.23*	*0.31*	*0.98*	*0.30*	*0.29*	*0.29*
**Number of Observations**	1451	165	1286	1286	1224	1451	165	1286	1286	1224
**Competence Mean**	0.00	0.64	−0.09	−0.09	−0.13	0.00	0.64	−0.09	−0.09	−0.13
**Competence Standard Deviation**	1.24	0.98	1.24	1.24	1.24	1.24	0.98	1.24	1.24	1.24
**Regression***r*^2^	0.08	0.15	0.06	0.26	0.24	0.35	0.47	0.34	0.38	0.36

Four findings are particularly noteworthy. First, having an MBBS degree is associated with 0.56 standard deviations higher knowledge scores (Table 3), which corresponds to a 10 percentage point increase in the likelihood of correct case management for our vignette cases. Part of this advantage reflects a larger number of non-MBBS providers in states where knowledge is lower overall and therefore the MBBS advantage is reduced once we include state fixed effects in Columns 6–10 (Appendix Figure A8). Although it seems from these estimates that within states there is little difference in the knowledge of MBBS providers who have chosen to locate in rural areas versus other providers, we note that the very small number of MBBS providers in the village sample cautions against drawing definitive conclusions.^9^

Second, there is little association between provider age and knowledge; the coefficient is qualitatively small and never statistically significant. Given the substantial age variation in our sample, this implies that the quality of new entrants is similar to those who entered 30–40 years earlier. This suggests limited improvements in medical education over time, or that older cohorts have been able to learn on the job, keeping abreast of improvements in medical knowledge over time. Given that there is no re-certification requirement in India and few opportunities for continuing medical education in rural India, the former may be the main explanation.

**Fig. 5:**
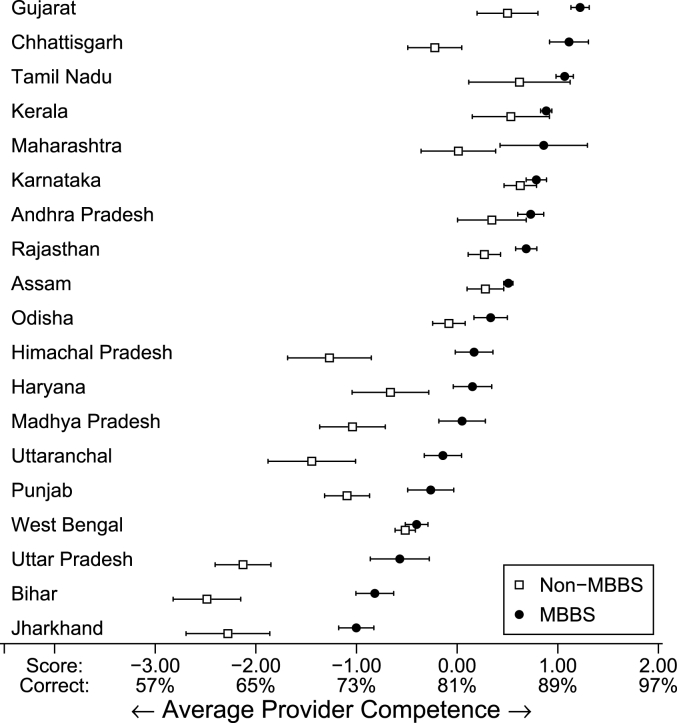
Provider knowledge by degree qualification and state. Notes: This figure reports the competence scores of the full sample of providers who participated in vignettes testing. It displays both the distribution of scores for providers who were identified as having an MBBS degree in each state (N = 1738) as well as the distribution of scores for providers who were not identified as having an MBBS degree (N = 2091). The mean provider competence corresponds to a score of zero and the standard deviation is 1. States are ordered by the score of the mean MBBS provider in the vignettes sample. *Data source:* village provider census and survey; village and PHC/CHC vignettes sample.

Third, based on the regression R-squareds in Columns 1 and 5, provider characteristics and district SES account for just 8% of the overall variation in knowledge (5% when district SES is excluded) compared to the 27% of the overall variation in our data that arises due to differences across states. This has fundamental implications for policy. Fig. 5 plots the mean knowledge score for MBBS and non-MBBS providers for every state in our sample with 95% confidence intervals; we also provide the proportion of correctly managed vignettes expected for each knowledge score. As Fig. 5 shows, the average MBBS doctor in Jharkhand and Bihar is about one standard deviation below the nationwide average, while the average MBBS provider in states like Tamil Nadu and Gujarat is one standard deviation above the national average. This difference translates into a 20–30 percentage point increase in the likelihood of knowing how to correctly manage a case.

More importantly, Fig. 5 also shows that the medical knowledge of IPs is highly correlated with that of MBBS providers in the same state. Informal providers in high-performing states routinely outperform MBBS providers in low-performing states and in fact, in these states, the difference in quality between MBBS and non-MBBS providers is small. This trend implies that across Indian states, equal qualifications do not imply equal quality. The knowledge of an informal provider in Gujarat or Tamil Nadu is substantially higher than that of a fully-trained MBBS provider in Bihar or Jharkhand.

Finally, the knowledge of providers in the public and private sector follow similar patterns – states where public providers are more knowledgeable are also those where private providers are more knowledgeable and this relationship continues to hold at the level of the district once we include state fixed-effects (Table 3, Columns 7 and 8). Across all explanatory variables, the competence of providers in the public sector is the one that is most strongly associated with the knowledge of private providers and IPs. This relationship remains equally strong when we include state fixed-effects, suggesting that even within states, districts where public sector providers are more knowledgeable are also those where the private sector providers are more knowledgeable. Socioeconomic development in India does not crowd out informal providers: it increases their knowledge, a fact that may explain why IPs do not disappear as states become richer.^10^

#### 4.2.1 Where are the good doctors? An example of what quality variation implies

**Fig. 6:**
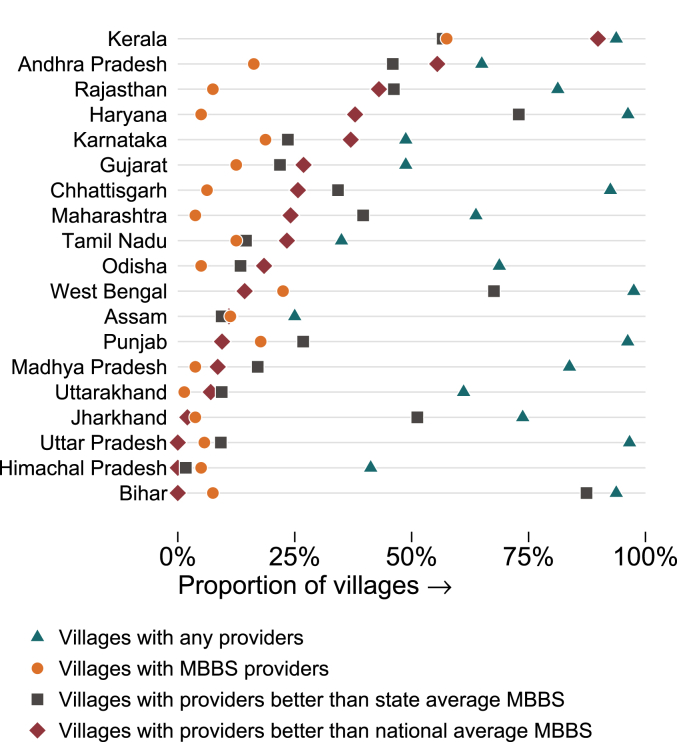
Availability of quality care in villages. Notes: This figure illustrates the effective availability of care across villages in each state based on the counts obtained in our survey (triangles) and on three different alternative quality definitions. In the first alternative definition (dots), we illustrate the proportion of villages in each state with an MBBS provider. In the second (squares), we illustrate the proportion of villages that have a provider whose competence score was greater than the score of the average MBBS provider in that state. In the third (diamonds), we illustrate the proportion of villages that have a provider whose competence score was greater than that of the average MBBS provider across the entire sample. The average MBBS provider is the provider with a score of 0.00 on the vignettes; this provider on average got 81% of vignette treatments correct. States are ordered by their ranking in the final measure. *Data source:* village provider census and survey; village vignettes sample.

One key finding in our data is the enormous *variation* – both across states and within villages – in the knowledge of health care providers. To expand on what this variation implies, Fig. 6 shows the fraction of villages with access to a “high quality” provider under three different definitions (as compared to access to any provider). We start with the status quo: Providers are defined to be high quality if they have an MBBS degree. As shown previously, this implies that only 11% of villages have access to quality medical care, with wide variation across states. In the figure, the fraction of villages with an MBBS provider is represented with a circle.

Alternatively, suppose that we define a “high quality” doctor as one whose knowledge is above the mean knowledge of MBBS doctors in the *entire* Indian sample. As we have shown in Table A3, such a standard implies that providers will know how to correctly manage TB and diarrhea 80% of the time and preeclampsia 67.1% of the time. Despite the low bar, in Himachal Pradesh, Bihar, and Uttar Pradesh, no provider in our sample met such a national cutoff. In contrast, several states in the South have at least one provider who is higher quality than the national MBBS-average cutoff in many villages, and in these states, availability of a "high quality doctor" is higher than using a measure based just on the availability of MBBS doctors.

A third possibility is that we redefine access to high quality as access to a provider with at least the average knowledge of MBBS providers *within* the state. We have chosen the mean knowledge score of MBBS providers in the state as a cut-off; if we assume that current MBBS levels of quality are consistent with a revealed preference for quality given the costs of training in the state, such a cutoff represents a useful starting point. If qualifications determined quality (so that the least knowledgeable MBBS was still more knowledgeable than all IPs), we should expect that very few villages satisfy this criterion: by construction, half of existing MBBS providers and all IPs in each state will be below the quality cutoff. Instead, we find a substantial increase in every state except Kerala, Assam, and Himachal Pradesh. This is because most villages have multiple IPs and the quality of these IPs varies widely, with many IPs outperforming the average MBBS in the state. The three states of Kerala, Assam, and Himachal Pradesh are the only ones in India where there are not at least as many non-MBBS providers who are higher quality than the average MBBS in the state as there are MBBS who are worse. By this definition at least, most Indian villages already have access to a high quality provider – it is just that they do not have any formal medical training.

### 4.3 The Two Indias: putting together cost and quality

Our measurements now allow us to construct a composite picture of health care in rural Indian in terms of per-visit costs and quality, which we continue to proxy for with provider knowledge. We compute average quality in a village as the quality of each provider weighted by the markets shares of their clinics. The per-visit cost depends on the market share of each clinic, fees of private sector providers and the wages of public sector doctors. In particular, public sector doctors are paid on a fixed salary while private sector providers charge a fee per patient. Therefore, the per-visit cost in a village can be written as $C_{i}=\frac{Wage_{public}}{Total\cdot Patients}+\left(Fee_{private}\times Share_{private}\right)$, where $Wage_{public}$ is the total wages in the public sector, $Fee_{private}$ is the average per-patient fee in the private sector and $Share_{private}$ is the share of total patients in the private sector.^11^

**Fig. 7:**
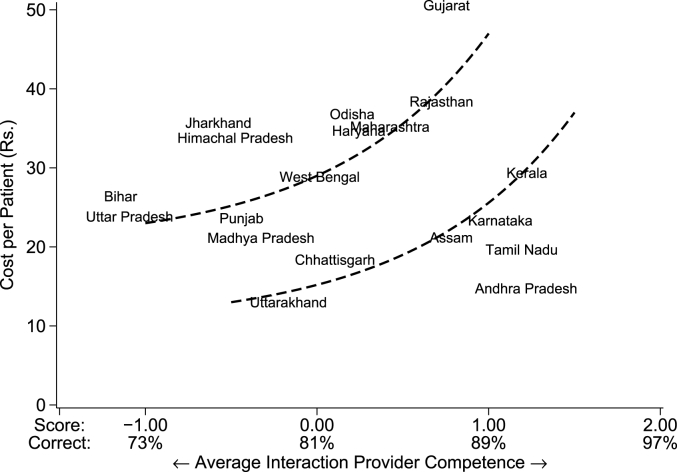
Average cost and quality per patient interaction. Notes: This figure plots the cost in Indian rupees per visit seen against the average caseload-weighted provider competence for each state. The cost per visit in the public sector is calculated as the sum of the total public sector wage bill using reported monthly salaries from all providers and dividing this total by the total number of patients seen each day multiplied by 20 working days per month. For the private sector, it is the greater of total reported medical income divided by the number of patients seen per day times 20 working days; or the self-reported fee per patient. The reference lines are added manually for illustration purposes. The mean provider competence corresponds to a score of zero and the standard deviation is 1. *Data source:* village provider census and survey; village and PHC/CHC vignettes sample.

Fig. 7 shows the cost and quality in every state and justifies our “Two Indias” framework. The vertical axis shows cost per patient in Indian rupees, while the horizontal axis shows the average quality of the provider seen by primary care patients. Appendix Table A4 shows state-wise estimates for each of these variables.

The significant variation in per-patient price and quality is immediately obvious. The per-visit cost is much lower in Andhra Pradesh compared to Gujarat. In Bihar, the average patient quality is at the bottom of the quality distribution, two standard deviations below states like Karnataka and Kerala. Most striking about this variation is that Indian states divide into two very distinct groups. The first group (Group 1) includes states like Uttar Pradesh and Bihar, but also Gujarat and Orissa, which are arguably on the same frontier where higher quality (as in Gujarat) comes at higher costs. The second group (Group 2) includes the four Southern states of Kerala, Karnataka, Tamil Nadu and Andhra Pradesh, but also Chhattisgarh and Uttarakhand, which have achieved higher levels of quality at lower per-patient costs than similar-performing states in Group 1. If we were to think of the price-quality trade-off as a production possibility frontier, which we (heuristically) illustrate in the figure, the data suggest that these two groups correspond to two different such frontiers in India. Group 2 states appear to be operating at a significant quality *and* budgetary advantage relative to Group 1 states. As we illustrate next, this idea of “Two Indias” is fundamental to thinking about health policy.

### 4.4 The Two Indias: thought experiments

The density of health markets at the level of the village and the remarkable stability of the informal sector across states has significant ramifications for ongoing debates in Indian health care. With the majority of primary care delivered by IPs, the country simply cannot afford to outlaw 70% of its primary care workforce without a replacement strategy. Several such replacement strategies have been proposed and we use two thought experiments to show how the variation in our data introduces complexities for any policy change. Since they assume no behavioral responses among providers and pre-determined responses among patients, these must not be interpreted as ex-ante estimates of policy changes, but simple illustrations of what state- and village-level variation in quality and price may imply for policy.^12^

#### 4.4.1 Making AYUSH providers public

One thought experiment examines a proposal to further integrate AYUSH providers into the public workforce; this, for instance, is one part of the primary care strategy embodied in India's new plan called Ayushman Bharat. The effect of this policy depends on (a) the pre-existing access to AYUSH providers in the *private* sector and (b) the relative earnings of AYUSH providers in the public and private sector.^13^

**Fig. 8:**
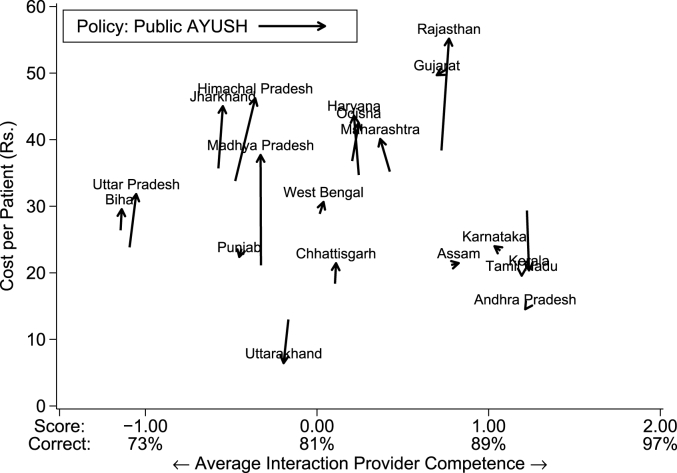
Average cost and quality (AYUSH counterfactual). Notes: This figure plots the cost in Indian rupees per visit against the average caseload-weighted provider competence for each state under two scenarios: the data as observed, and a counterfactual in which all private AYUSH providers are shifted to the public sector. We reassign an additional 50% of the private AYUSH patient demand from private non-MBBS providers to the now-public MBBS. We adjust the monthly costs of these AYUSH to equal the average state monthly salary for existing public AYUSH providers; we adjust the monthly costs of the private non-MBBS providers proportionally to their new share of patients. The mean provider competence corresponds to a score of zero and the standard deviation is 1. *Data source:* village provider census and survey; village and PHC/CHC vignettes sample.

Fig. 8 shows that this policy increases costs among Group 1 states due to the salaries necessary to support the new public workforce. The effects on quality are limited at best because the quality of AYUSH providers is simply not that different than other non-MBBS providers. In some states, like Rajasthan, Madhya Pradesh, Jharkhand and Himachal Pradesh, the relatively high salaries of existing publicly-employed AYUSH providers means that the policy is expensive to implement. In most, the private AYUSH who would be transferred to the public sector are similar quality to the current providers visited by patients, leaving average quality unchanged. In contrast, Group 2 states see almost no change in either price or quality. In these states, the salaries of AYUSH in the public sector as well as their quality is very close to non-MBBS providers in the private sector and therefore moving them from one sector to the other does not substantially change the equilibrium.

#### 4.4.2 Expanding the public sector

A second thought experiment looks at an implausibly massive expansion of the public sector, where we assume that the government can hire enough MBBS providers to staff every village currently without a public option. While the actual proposal of expanding to every village is not actively discussed, due to its very high cost, at the very least policymakers assume that such a policy will dramatically increase quality. We examine a naive version of this policy, where the government bears zero cost of training new MBBS providers; this extreme assumption helps us provide a lower bound for the costs of such a policy.^14^ Because this policy requires the government to move into new villages, the effects are even less predictable as it further depends on the attributes of the new villages that receive these PHCs relative to the villages that already have them. If, for instance, these villages are much smaller, it also implies that the costs will be commensurately higher due to lower patient loads.

**Fig. 9:**
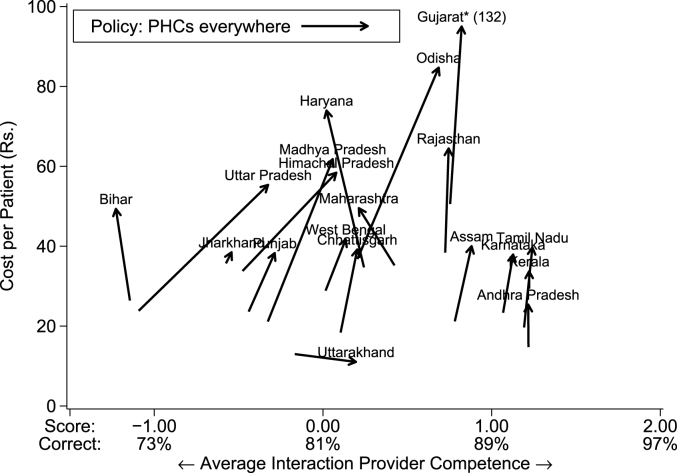
Average cost and quality (PHC counterfactual). Notes: This figure plots the cost in Indian rupees per visit against the average caseload-weighted provider knowledge for each state under two scenarios: the data as observed, and a counterfactual in which a public-sector MBBS provider is placed in every village that does not have one in the data. We reassign patients within every village to match the distribution across provider types as observed in each state in the average village that has a public-sector MBBS provider. We set the monthly costs of these MBBS providers to equal the average state monthly salary for existing public MBBS providers; we adjust the monthly costs of all private sector providers proportionally to their new share of patients. The mean provider competence corresponds to a score of zero and the standard deviation is 1. (*Under the simulation counterfactual, the cost per patient in Gujarat is Rs.132; we cap the illustration to preserve scale on the figure.) *Data source:* village provider census and survey; village and PHC/CHC vignettes sample.

Fig. 9 shows that in Group 2 states, with already well-developed public sectors, there are again much smaller changes: costs increase somewhat, but quality is mostly unchanged. In Group 1 states, there is a wide range of possible outcomes in terms of quality improvements, but the costs are always significantly higher than in the Group 2 states due to the high salary costs of MBBS providers and their low patient load. In Haryana, Maharashtra, and Bihar, the low quality of the public providers combined with the low patient demand means that the policy is expensive with an *adverse* impact on quality. In contrast, costs increase substantially in states like Uttar Pradesh and Madhya Pradesh, but so does quality. The striking point from this thought experiment is that even if India was willing to expand MBBS providers through massive public sector recruitment to every village, the quality effects remain highly uncertain in Group 1 states and could even decline as patients move away from better quality (but fee-charging) private providers to free public provision, albeit with lower quality.

#### 4.4.3 Summary

Across both thought experiments, Group 2 states are minimally affected. These states have relatively robust public sectors that closely match the private sector in per-patient costs and quality. Shifting providers from one sector to the other does not dramatically change costs per patient, even if it can decrease out-of-pocket costs. In Group 1 states, the impact of the simulated policies is both large and variable. Costs are not controlled, and the quality impacts depend heterogeneously on the specific composition and distribution of providers and patients in that state's rural sector.

The policy environment thus seems to be cursed by a structure of “the best and the rest”. In some states, better transportation networks and population density have created the economies of scale necessary to support high-quality provision of care for most of the population. In those states, health outcomes seem to be structurally robust to intervention; there is an equilibrium where both cost and quality are competitively balanced across sectors and adjustments to patient choice on the margin have little effect on either. In the others, economies of scale and integrated markets do not yet exist and therefore costs and qualities remain extremely diverse across villages. These health markets are unstable, and policy changes can have extreme impacts, but the consequences are unpredictable and depend critically on the precise composition of the market and the dispersion of patients across villages in terms of access and quality.

## 5 Discussion of limitations and conceptual issues

In this section, we first discuss data limitations that we (now) know how to solve and we could repeat this study without replicating these mistakes. We then discuss conceptual issues without an easy answer. These are problems that need to be further studied if we are to make progress in this important arena.

### 5.1 Data limitations

**Incomplete information.** One fundamental insight from this work is that we need to conceptualize the Indian environment as health care *markets* with multiple providers competing for patients. In this environment, sampling among health care *providers* can be very misleading. First, if high- and low-quality providers co-locate in markets, then averages based on random provider samples will suggest variance across villages where there is none. Further, differences in the total number of low-quality providers would cause average quality to decline in a state if there are more low quality-providers – even if the same number of available high-quality providers are available as in another state. But patients may, in fact, be better off by having access to one high and one low quality provider rather than only one high quality provider. Consequently, provider-average measures of quality may not be useful metrics for the welfare of patients.

This requires further research on the construction of markets, which we discuss below, and the sampling of providers within markets. Specifically, once markets have been constructed, all providers in the market should be covered in the survey as in Das et al. (J. Das, Holla, et al., 2016) Such a strategy allows us to compute multiple quality statistics that include the mean, but also additional characteristics of the distribution such as the quality of the best provider. Combined with data on market shares, we believe this to be the most policy-relevant data for understanding the Indian context. The current study implemented this strategy for measuring provider availability, but then returned to a sample of providers for quality estimation.

**Attrition.** The two-stage sampling strategy also led to non-completion of the vignettes for 40% of the sampled providers. Appendix C examines non-completion in greater detail. The conclusions from this analysis are two-fold. First, we see that those who did not complete the vignettes had greater education levels, more permanent occupations as health care providers, higher caseloads and fees, and more time reported with their patients. These differences are small in magnitude but statistically significant; they all suggest that doctors with more regular and established practices were more likely to be located and to participate. To the extent that these characteristics are correlated with levels of knowledge, our quality measures are biased.

In order to obtain a better picture of the potential size of this bias, we machine-select those covariates that best predict completion of the vignettes model and regress vignettes performance on this set of covariates. Substantial bias based on observables would require some covariates that *both* affect the likelihood of completion and the knowledge of the provider; in fact there are few such covariates and when we correct for non-response based on observed characteristics, there is little change in key estimates, as shown in Appendix Table A7. We conclude that corrections for missingness based on observables will not substantially alter our mean estimates; non-response based on unobserved characteristics that are correlated with knowledge cannot be assessed in these data. Beyond estimates of the mean, missingness will almost surely affect estimates such as the quality of the *best* providers in each village. Consequently, our thought experiments on (for example) quality regulation may be biased since we will almost surely have missed some high-quality providers in each state.

We believe that both the problems of inference from village-level samples (rather than censuses) and attrition can be fixed with an adequately resourced data collection exercise in the future.

**Outdated information.** Our data collection was finalized in 2010, and there could have been substantial changes to the rural landscape since then. We attempted – and failed – in our attempts to intelligently extrapolate some our data to a more recent year, so the best we can do is speculate on what may have changed substantially in the intervening 9 years.

First, there is no reason to believe that these changes will have affected the composition of the rural workforce, as more recent studies from specific geographies point to similar patterns. See, for instance, Gautham and others for the states of Uttarakhand in North India and Andhra Pradesh in South India; Das and others in West Bengal and most recently, Shailaja Chandra and others in Uttar Pradesh (Chandra and Bhattacharya, 2019; J. Das, Chowdhury, et al., 2016; Gautham et al., 2014).

Second, a simple calibration suggests that changes in the levels of knowledge are unlikely to have been substantial during this time. If the age distribution is stable, the quality improvement over the last decade will be similar to reducing the age of all providers in the current distribution by 10 years. The only missing data for this computation is the quality of new entrants, which we can bound using the lower 95th confidence interval on the age estimate in the regression of knowledge on age (0.01 SD per year). When we do so, we find that quality could have improved *at most* by 0.1 standard deviations at the national level. The reason for this small change is that the correlation with age is close to zero (and statistically insignificant): There is little evidence that new cohorts of medical graduates are better trained than those who graduated decades ago.

Third, what has almost certainly changed since 2010 are the prices of services in the private sector and wages in the public sector, both due to inflation and changes in the demand for health care in a period of substantial growth. The relative per-patient cost of a primary care visit in the public versus private sector could have also changed due to the revision of doctors’ salaries and the public pay scale in several states. For instance, the Liver Foundation provided us data from patient observations among informal providers in 5 states in 2017, and we also have data from West Bengal from our own studies (J. Das, Chowdhury, et al., 2016). Fees in these data are 3–4 times higher based on observation, whereas inflation alone would have led us to expect a doubling of fees during this time. We cannot say whether this reflects a genuine increase in prices, or is a difference in the prices obtained from clinical observations versus provider self-reports.

Although we expected a lack of data on private sector fees, what is truly surprising there is also no data available on the salary of public sector providers in different states. The most updated published research is George and Rhodes (2017), who base their information on a study for the public sector and from the careers information site www.naukrihub.com. The sampling and provenance of this information remains unclear. Even trying to collect updated information from each state on salary *scales* will not be informative as the salaries of providers in rural areas are only weakly predicted by mean salaries in the state and their observed characteristics. There is clearly an urgent need to collect per-patient cost data on a regular basis in the public sector, but for the purposes of our analysis, presenting the data from 2010 is a safer choice relative to extrapolating on the basis of insufficient information.

What we found interesting from the limited recent research is that, while the overall fees and salaries are clearly different, the patterns across states appear to be similar to our data from 2010. As a result, the cost implications of various policy proposals will be quantitatively different now, but we believe that the overall patterns and presented here will remain qualitatively similar.

### 5.2 Conceptual issues

**Construction of markets.** Even with a provider census within sampled villages, the construction of markets remains a challenge. In our Madhya Pradesh study, two-thirds of the care was sought within the village, and typically, there was one additional large cluster of providers outside each village, usually on the highway. Consistent with studies of bypassing, Das and Mohpal (2016) have demonstrated a distance-quality trade-off, with the quality of visited providers increasing in the distance from the household (Das and Mohpal, 2016). As Appendix Figure A3 shows, this bias is not constant across states. Our household survey showed that primary care visits in the states of Chhattisgarh, West Bengal and Kerala are predominantly within the village – in these cases, our market-share weighted estimates of quality, which we use for our simulations, are reasonable approximations. However, there are several states where the share of visits within the village is below 60%; in Tamil Nadu, it drops to 23%, which is consistent with our observation that fewer villages have their own providers. For the states of Andhra Pradesh, Himachal Pradesh, and particularly Tamil Nadu, our weighted quality estimates are likely a lower bound. How to address this problem in a state like Tamil Nadu is unclear. Our team's experience was that with a better transport system, people often take the bus to the town for their health care needs, so that urban and rural availability and quality may be similar. Larger catchments substantially increase the cost and complexity of surveys such as these and further progress may require administrative data, for instance, through insurance claims.

**Defining provider quality.** We use knowledge of the correct treatment in vignettes as our measure of quality. We have shown previously that knowledge represents the maximum quality that patients can receive, and there is a usually a significant gap between clinical practice and knowledge (Das and Hammer, 2014; Das et al., 2008). Although this should not bias the variation across states as practice and knowledge are strongly correlated, it does imply that most of our estimates are upper bounds of the care that patients actually receive. We have also abstracted away from the use of unnecessary treatments, but these may be important in their own right. For instance, the widespread use of antibiotics and India's high rates of anti-microbial resistance are a particular concern. If correct management also implied less use of unnecessary medicines, relative rankings remain unchanged. Unfortunately, Appendix Figure A2 shows that providers who were more likely to give the correct treatment were *equally likely* to give unnecessary antibiotics, driven to a large extent by the greater use of unnecessary antibiotics among fully trained MBBS providers. This pattern differs from a common wisdom that informal providers are responsible for antimicrobial resistance in India and raises the difficult problem of how to weight treating the patient correctly versus the incorrect use of antibiotics. Given the patterns in our data, the inclusion of unnecessary medicines substantially complicates the picture and deserves full attention in its own right.

**Behavioral responses.** Finally, our thought experiments do not take into account behavioral responses. Consequently, they are not meant to provide specific policy guidance but rather to highlight that under certain behavioral assumptions, these policies can yield a variety of results. The broad point remains that these policies are currently being advocated without estimates of key elasticities in the rural market for care, largely driven by models that do not account for the variation in quality and price within each market. This limitation therefore reflects a broader problem with policy proposals in this area.

## 6 Conclusion

Our data highlight multiple features of the primary care environment in India that are at odds with current perception among researchers and policy makers. We first clarify that 86% of full-time rural health care providers in India are in the private sector and 68% have no formal training. They are, therefore, operating illegally, and outside the ambit of health policy as it currently stands. Put simply, most primary health care in rural India is in the hands of providers who don't legally exist. This explains why, despite repeated assertions since at least the J.P. Bhore committee in 1946 that the Indian government will remove informally trained providers from the primary care market, implementing any such policy has proven to be extremely difficult.

Across-state variation shows that the dominance of IPs does not mechanically decline with socioeconomic development. The idea that “as states grow richer and the public sector becomes better, IPs will vanish” simply does not hold in our data. Instead we find that as states develop and (especially) as the quality of public sector providers improves, IPs themselves become more knowledgeable, keeping up with their competitors in the public and private sector. The fundamental point that equal qualification does not imply equal knowledge across states is germane for policy discussions; for instance, it feeds back into the result that responses to policy changes in Group 1 states are difficult to predict ex ante.

It also has implications, for instance, for the proposed National Exit Test, advanced by the central government through the National Medical Commission Bill, 2019. According to this proposal, the granting of medical degrees by individual institutions would be replaced by a centralized examination. But what should these cutoffs be? As we have shown in Fig. 6, if the cutoff is at the national level, in many states it may be that no MBBS doctors pass the test. An alternative is state-level cutoffs, but then it is not clear what problem the test will solve, as many health care providers in the informal sector might already meet state-level quality standards.

Having cast doubt on several policies, we highlight three areas where we need to think differently for progress to be made.

We need to better understand and communicate to Indian and global policymakers the fundamental “aggregation problem” in rural health care. In sparsely populated rural locations, the maximum patient load of a clinic is severely limited by geography. Because of the structure of transport networks, larger health facilities in central markets and cities are easier to access than the next nearest village. People may visit the clinic in their village, but if they have something more serious they will go to the highway, and if they have something really major they will take a bus and go to the city. But people will almost never travel from one village to another, because it usually requires going to the highway anyway and then taking another peripheral road out into another village. A high-quality provider who is posted to a village will spend a much smaller portion of the day seeing patients compared to a provider in a district hospital.

In this world it is impossible for any rural clinic to expand its catchment population beyond the village it is located in. Paying full-time salaries to staff in clinics that are operating with substantial unused capacity is an extremely expensive way to provide care in these areas, as our cost calculations for the public sector demonstrate. This problem poses an especially challenging trade-off as additional staff for crowded urban clinics are likely to be used to capacity, whereas the same additional staff in rural clinics will remain idle. Therefore we need to promote a clear understanding of why spatial features make the per-patient costs of public primary care so high in rural areas, allowing for better decision making about capacity allocation. Indeed, given the patterns of usage, better roads and public transport that increase access to higher-quality providers in urban areas may be a more viable policy than providing (mostly empty) clinics in sparsely populated rural areas (Aggarwal, 2018).

The second point is a simple application of supply and demand. In markets with quality and price, demand shifters will typically increase quality and price, while supply shifters will increase quality and decrease price. The “Two Indias” that we have identified differ in both quality and price, and in our data, Group 2 states enjoy higher quality at lower per-visit costs. Part of this depends on wage-setting in the public sector and who is posted to rural areas. But part of it also depends on the supply of doctors. It is striking that the Group 2 states are also those where medical colleges and nurse training institutions are located.

Ajay Mahal and Manoj Mohanan (2006) were among the first to point out how the growth of private medical colleges was exacerbating existing geographic inequities through the historical placement of public institutions (Mahal and Mohanan, 2006). Sabde and others (2014) have carefully mapped the growth of medical colleges in India and they further confirm the substantial disparities across what we have called the Group 1 and the Group 2 states: “*In our study, the corresponding proportions in the aforementioned northern and southern provinces were similar: 11.8*% *and 56.3*%*, respectively, in 2012. In general, the southern provinces have had better socio-economic indicators compared to the northern provinces.*” (Sabde et al., 2014) In our data, only 20% of providers are working outside the district they were born and an even smaller fraction (7%) outside the state they were born. Given the fragmentation of the labor force, it could be that the availability of medical education has a large effect on the entire primary health care market. If so, greater investments in medical education for the Group 1 states would offer a clear policy path moving forward.

The final observation is that the existing dense network of rural health care providers needs to be understood as a market. Since it is primarily a private market, forces of price and choice are core determinants to the actual quality of care delivered to patients, and understanding these provider networks in this context is an open field for study. We cannot emphasize enough that the complexity of health markets needs to be embraced, rather than shied away from, and once we allow for the possibility of patient choice, multiple policy opportunities become available. For instance, our results suggest that using a national exit test with equally high standards in all states will drastically reduce access in poorer states. However, this does not preclude the option of using such examinations to provide a standard score for all examinees. A standard score will allow states to set their own cutoffs, adjust cutoffs for existing stocks versus new entrants and enhance the portability of medical degrees across states.

What we now need to understand is (1) how well care-seekers are able to identify and afford higher-quality service as they need it; (2) how well provider prices accurately reflect the quality of their practice; (3) how responsive the overall quality of services is to local socioeconomic status and (4) the link between the availability of medical education and the quality of the primary health care workforce. If higher quality providers are recognized by the population and better training sets off a virtuous cycle that improves the quality of all types of providers, this opens up multiple possibilities moving forward.

## Authors contributions

Jishnu Das: Writing- Original draft preparation, Funding acquisition, Data curation, Supervision, Formal analysis, Methodology; Conceptualization; Benjamin Daniels: Writing- Original draft preparation, Visualization, Software; Formal analysis; Monisha Ashok: Data curation, Writing- Reviewing and Editing; Eun-Young Shim: Data curation, Writing- Reviewing and Editing, Formal analysis; Karthik Muralidharan: Data curation, Writing- Reviewing and Editing, Funding acquisition, Supervision.

## Appendix A: Supplementary data

Supplementary Appendices Multimedia component 1

## Footnotes

1 The most comprehensive of these studies is De Costa and Diwan (2007) in the state of Madhya Pradesh (De Costa and Diwan, 2007). Other examples include Das and Hammer's work in Delhi, Das and Mohpal's work in Madhya Pradesh, Gautham's surveys from Andhra Pradesh and Uttarakhand and Rao et al.‘s surveys from Chhattisgarh (J. Das and Hammer, 2007a; J. Das, Holla, et al., 2016; Gautham et al., 2014; Rao et al., 2013).

2 As Anand and Fan (2016) have shown, 31.4% of these providers were educated only up to secondary school and therefore *cannot* have been formally trained. As general levels of education have increased, a significant fraction of providers with secondary education may have no formal training, making it impossible to ascertain the actual qualifications of providers that rural Indians access using census data (Anand and Fan, 2016).

3 Based on census data, Anand and Fan (2016) estimate 50.6 Allopathic doctors (qualified and unqualified) and 163 health care providers (including nurses, midwives and pharmaceutical staff) per 100,000 population (Anand and Fan, 2016). By contrast, de Costa and Diwan's (2007) statewide enumeration counted 41 qualified doctors and 156 qualified non-doctors (mostly nurses and midwives) per 100,000 population. They also counted an additional 238 trained birth attendants and untrained providers per 100,000 population, more than doubling the total number of health care providers in the state (De Costa and Diwan, 2007). The combined total of 435 health care providers per 100,000 population discovered through the survey puts Madhya Pradesh – one of India's poorest states with high child mortality – just below Greece at 6th place in the World Development Indicators ranking of doctor density around the world (http://nationmaster.com/NationMaster.com, 2019).

4 Exceptions are Uttarakhand where we sampled 9 districts (72 villages) and Uttar Pradesh, where we sampled 11 districts (88 villages). In our sampling frame from 2003, Uttarkhand had only 9 districts and so the additional district was assigned to the much larger neighboring state of Uttar Pradesh. Finally, our study was completed prior to the division of Andhra Pradesh into Andhra Pradesh and Telangana. To keep the results consistent with the sampling, we refer to these two states as Andhra Pradesh, but readers should be aware that the sample is drawn from both states.

5 To capture all the medical service providers in a village, enumerators first conducted “Participatory Resource Assessments” with households at multiple locations (at least three) within each village to obtain a list of all medical service providers within the village boundary. After enumerators conducted this assessment they visited each provider in the list and asked about other providers in their village, in case they had been missed.

6 We exclude paramedical staff, who account for 83% of all public providers but are mostly responsible for preventive care with a very small share of primary care visits (Appendix Fig. 1 presents the statewide distribution of paramedical staff).

7 The 20% non-completion of the sampled providers implies that these could be lower bounds of the true accessibility to different types of providers. However, since this represents “business as usual”, it is also reasonable to believe that on an average day or week this is the true accessibility in the village once provider absence is taken into account.

8 In the 10% of cases where there are multiple providers in a clinic, we assume that all providers report seeing the same sample of patients, and therefore apportion the mean number of patients reported among the number of reporting providers. Total workload is then computed as time per patient multiplied by patients per provider day.

9 Our total sample of 62 private sector MBBS providers is mostly located in the better performing states of Kerala, West Bengal, Karnataka and Andhra. One alternative is to use the full sample of village and PHC/CHC vignettes to estimate the within-state MBBS coefficient. When we do so, we find that the MBBS coefficient with state fixed effects is 0.82 SD, although the MBBS advantage varies across states. However, this estimate is based primarily on public sector providers who may or may not be located in rural areas and is therefore not strictly comparable to the estimates in Table 3. See Appendix C for associated tables and figures.

10 A more subtle pattern is that the association between district SES and the knowledge of private providers disappears when we include the competence of public providers in Column 4. However, the association does not change once state fixed effects are included. This is because at the *national* level, higher SES districts have more knowledgeable public providers, but at the *state* level, this correlation disappears. See AppendixFigure A7.

11 For cost computations we use self-reported fees or reported medical income in private clinics and the total salary cost of the facility divided by the patient-load for public clinics – equivalent to the total health expenditure divided by the number of patients seen. Again, for the public sector this is a lower bound of the cost per visit as we exclude the costs of infrastructure, equipment and administration.

12 For instance, we assume that providers will not choose to relocate or re-skill in response to the policy change or that moving providers from the private to the public sector will not affect their quality. The last is an assumption that we know to be incorrect as our previous work demonstrates that quality is higher for the same providers in their private compared to their public clinics (J. Das, Holla, et al., 2016). Why do them at all? We know that effects of any policy depend on (a) demand and supply-side behavioral responses and (b) the preexisting situation. There are no estimates for the former in the literature for India, and this is the first paper to provide a detailed picture of the latter. At the very least, therefore these data illustrate how policy changes can have varied effects across Indian states.

13 We examine this policy by (a) moving all existing private AYUSH providers from the private in the public sector in the same village; (b) adjusting their cost to the average public AYUSH monthly salary in that state; (c) assuming that their patient loads will increase by 50% because they will become free at the point-of-use for patients and; (d) assuming that these new patients will be drawn equally from each non-MBBS private sector provider in the village. We do not change provider availability or total patient demand.As proposed, the policy would also include two years of training for the AYUSH providers, but this is conceptually separate from expanding the number of AYUSH providers in the public sector. That is, if the training is evaluated and shown to be cost-effective, it should be provided regardless of whether a provider is in the public or private sector.

14 We assume that the market share of the public sector in these new PHCs will be identical to the average market share in villages that currently have a public sector clinic. We also assume that the quality of new public sector clinics will be equal to the state average in existing public MBBS providers. We then use the prevailing state average salary costs to determine the per-patient costs after this reform. We do not assume any new patient demand.
