# Supplementary material for: Two Indias: The structure of primary health care markets in rural Indian villages with implications for policy
Source: Soc Sci Med. 2022 May;301:112799. doi: 10.1016/j.socscimed.2020.112799 (PMC9188269; doi:10.1016/j.socscimed.2020.112799)
Supplement: Supplementary Appendices [file mmc1.pdf]

Two Indias: The structure of health care markets in rural Indian villages with implications for policy

Supplementary Appendices

January 23, 2020

Appendix A: Sampling and Survey Completion

The Medical Advice Quality and Availability in Rural India (MAQARI) Study was conducted in the 19 most populous states in India, except Delhi, which was not included because the study focused on rural India. The MAQARI study of quality and availability is intended to reflect a nationally-representative sampling strategy with weights appropriate to reporting the availability of providers to the *average person* living in a rural area. The MAQARI sample comprised two distinct sampling frames. In the first, a census exercise was conducted to identify all providers practicing in a representative sample of villages in rural India – the main Village Provider sample, which is used to calculate all availability, cost, and quality statistics at the village level in the main text. The study also included a representative sample of Primary Health Centers (PHCs) and Community Health Centers (CHCs), which are the central units of public health care system in India – the PHC/CHC sample. However, these centers tend to be located in denser and more urban areas. Therefore, this sample is used only for the construction of vignette scores and state-level knowledge measures in this study, and is not used for any calculation of costs or availability.

To construct these sample frames, a total of 10 districts were sampled from each state. To ensure broad geographical coverage, each state was divided into distinct sociocultural regions (SCRs), which served as strata for sampling districts and then villages. The number of SCRs in a given state ranges from three to eight depending on the size of the state. Once the state was divided into SCRs, the 10 districts were allocated across the SCRs proportional to population – so that larger SCRs had more sampled districts. The aim of the stratification was to ensure that the study is representative across all major social and cultural regions of the country. Once the number of districts was assigned to each SCR, districts were selected within the SCR with probability proportional to their population size. Since the study is on health care in rural India, districts with more than 60% urban population were removed from the sampling frame.

Districts and villages were randomly sampled using a population-based probability proportional to size (PPS) sampling method, on the basis of the 1991 population census. Villages were defined as under 100,000 residents in Kerala and under 20,000 residents for all other states. Within each sampled district, 8 primary sampling units (PSUs) were selected. Primary sampling units were PHCs or CHCs in the PHC/CHC sample and villages in the main Village Provider sample. Exceptions were Uttarakhand and Uttar Pradesh. Uttarakhand had only 9 districts, while Uttar Pradesh was the largest state in terms of population. Therefore, the Uttarakhand sample had 9 districts (90 PSUs) with the additional district assigned to the neighboring state of Uttar Pradesh, where we sampled 11 districts or 110 PSUs.

All analysis was weighted by the inverse of sampling probability, so the final estimates are nationally-representative on a population-weighted basis. Because sampling probability was proportional to population given a SCR, the sample represents the population in each SCR by construction. SCRs with larger population were allocated a larger number of sample districts; however, it is not an accurate reflection of differences in population size because of rounding error to obtain an integer. Moreover, sampling allocation was conducted within each state and does not reflect the differences in population across states. Therefore, we adjust for the population size by assigning the weight to each PSU (village or PHC/CHC), denoted by W_{PSU}, which is given by,

$$W_{PSU} = \frac{(SCR\ rural\ population)}{(Number\ of\ PSUs\ in\ the\ SCR)}$$

Note that all the PSUs in a given SCR are assigned the same weight. W_{PSU} is used in the calculation of the number of providers available per village analysis from the Village Provider sample. This choice of weight is designed to calculate the experience of representative population in rural India. Larger SCRs and villages are oversampled relative to those in smaller ones due to the PPS design.

To ensure the representativeness of the sample of medical service providers, enumerators first conducted a full mapping of all public and private providers in each sampled village. Enumerators conducted Participatory Resource Assessments (PRAs) with at least three households within each village to obtain a list of all medical service providers within the boundary of the village. After enumerators conducted the PRAs and compiled a list of providers, they visited all of the providers listed. These providers were asked about other providers in their village, and enuerators in turn added them to the village sampling list. All enumerated providers were then administered a Health Provider Census which included questions about their demographic characteristics, qualifications, patient fee, training, employment history, and various details of their practice.

20% of all listed providers did not complete the Health Provider Census (**Appendix Table A1**). Among them, 82 providers were recorded as having permanently left the clinic and they were dropped from the sample; 138 refused, and 21 had no reason recorded. The majority who did not complete the survey (618 providers) were temporarily away; therefore we believe the survey accurately reflects the average availability of providers as temporary unavailability is common among Indian providers. Anganwadi and Accredited Social Health Activist (ASHA) workers were also excluded from the sample because they are public health workers but not medical care providers (2,390 individuals). Providers with dental degrees and chemists or pharmacists were also excluded. The final village provider sample used in the analysis of the availability of village providers included including 4,335 health care providers, which we use to calculate the availability of medical care in the main text, and 1,622 paramedical staff, which we report here for completeness.

Appendix B: Medical Vignettes and Provider Competence Scores

Medical vignette administration

The provider listing resulting from the Health Provider Census was used as the sampling frame for follow-up surveys, which included medical vignettes for both public and private providers. Up to six providers per village were sampled for the follow-up surveys. If the village had six or fewer providers, all providers were sampled. If the village had more than six providers, one public provider was randomly sampled and then the remaining five were sampled from the pool of both public and private providers. Since there were fewer public providers, this sampling procedure ensured that at least one public provider was included in the sample if there was one in a village. If a village has many public providers because there is a CHC located in the village, up to four public providers were sampled. All estimates were re-weighted back to be representative of the provider type. In a PHC or CHC in a village, only doctors, nurses, Auxilliary Nurse Midwives (ANMs), and Multipurpose Worker (MPWs) were eligible to be administered vignettes. In sub-centers and private clinics, all providers were subject to vignettes. 4,464 providers were sampled for the vignettes and 2,704 providers completed.

All doctors, nurses, ANMs, and MPWs working in PHC/CHCs and all providers in sub-centers and private clinics in the sample were eligible to be administered vignettes. Vignettes were conducted by a team of two enumerators. For each vignette, one of the enumerators acted as the patient, while the other recorded all history and examination questions requested by the provider and furnished the results from the physical examinations carried out by the provider. The overall completion rate among PHC/CHC providers was 85% among MBBS and AYUSH providers and 45% among providers with no qualification who are mostly nurses, ANMs, and MPWs. The data used in this paper are for the sample of health care providers (that is, excluding those paramedical staff). Among public and private providers in the village sample who were selected for vignettes, the completion rates were 43% and 54%, respectively.

There is not enough information to conclude whether vignettes were missing at random, whether lower quality providers opted out of vignettes because they were not confident or higher quality providers opted out of vignettes because they had higher opportunity cost of participating. On the one hand, in PHC/CHC sample, the fact that the completion rate of qualified doctors was higher than other providers and anecdotal evidence reported by enumerators that many ANMs and MPWs refused to participate in the vignettes saying that they do not treat patients suggest that lower quality providers may have opted out of vignettes. On the other hand, the lower completion rates observed among qualified public doctors in the village sample suggests the other way around.

In the vignettes presentations, each provider was presented with three out of four hypothetical cases with specific symptoms. Providers were instructed to treat these cases as though these were their real patients and could ask any necessary questions, conduct tests or examinations, and recommend treatments. Four hypothetical cases used in the vignettes are provided below:

- **Tuberculosis:** A 40-year-old man comes to you. The man complies with all tests and medications that you recommend and will return to you if you require. The patient says, “Doctor, I have been suffering from a fever, cough, weakness, and

weight loss for the last month." (All providers)

- **Pre-eclampsia:** A 22-year-old female has come to you. As she walks in, you notice that her pregnancy is fairly advanced. The woman complies with all tests and medications that you recommend and will return to you if you require. The patient says, "Doctor, I have been having a splitting headache – it feels like my head is going to burst." (All providers)
- **Diarrhea:** A mother brings in an 8-month-old male child to you. The mother complies with all tests and medications that you recommend and will return to you if you require. The mother says "My child has been suffering from diarrhea for the last two days, and I do not know what to do." (Half of providers)
- **Dysentery:** A 25-year-old mother of a young child comes to you. She complies with all instructions, tests and medications that you recommend for her child and will follow up with you if you require. The mother says "Doctor, my 2-year-old child has been suffering from diarrhea for 2 days." This difference with the diarrhea case is that further questioning would reveal the presence of a fever and blood in the stool, suggestive of an infection. (Half of providers)

Each vignette case included an exhaustive list of questions that the doctor may ask with standard responses prepared for the enumerators to provide additional information to the provider. Enumerators recorded whether the provider asked questions or conducted examinations that are relevant to each case. Providers were also asked to pronounce diagnosis and recommend treatment for each case. Whether a provider prescribed correct medicine or gave a referral for each case were recorded. Every prescription was post-coded by a team of pharmacologists. For tuberculosis and preeclampsia, treatment was coded as being correct if a provider prescribed a correct medicine or if a combination of correct or partially correct diagnosis and a referral was given. For the diarrhea cases, treatment was coded as being correct if Oral Rehydration Salt (ORS) solution was recommended.

Provider competence score construction

The competence score variable was estimated using Item Response Theory (IRT) across a checklist of diagnostic questions for all vignette cases combined. This method creates a score which weights more difficult questions (those asked by the fewest providers) as more highly demonstrative of diagnostic knowledge, and eliminates redundancy between highly correlated items. The composite measure of provider quality is based on questions asked across all three cases and therefore represents an underlying generalized diagnostic competence score for that provider.

The same set of vignettes was administered with providers in PHC/CHC sample. This sample was used to calculate average knowledge scores for public providers across all districts; and to calculate state-level average competences for MBBS and non-MBBS providers overall. Again, all providers were administered tuberculosis and preeclampsia (hypertension in pregnant woman) cases, and a half of providers were administered a child diarrhea case and another half was administered a child dysentery case.

The distribution of scores in the full (combined) vignettes sample is illustrated in **Figure A2** and correlations are shown with: the correct management of the vignette case and the use of (unnecessary) antibiotics in the vignette case. Vignette weights

are derived from the survey design, and further adjustment was made to account for heterogeneous vignettes completion rates across provider types and regions. Let us define a variable V_{prov} for each provider of a given (s,p,m) category in a given PSU/district, where,

$$V_{prov} = \frac{Number\ of\ all\ providers\ in\ the\ PSU/district\ with\ the\ same\ (s,p,m)\ in\ the\ sample}{Number\ of\ providers\ in\ the\ PSU/district\ with\ the\ same\ (s,p,m)\ who\ completed\ vignettes}$$

V_{prov} was computed separately for PHC/CHC sample and village sample. Suppose that there are three doctors with MBBS degree in a CHC district in PHC/CHC sample and the two of them completed the vignettes. Then, each of the doctors who completed vignettes is assigned V_{prov} = 1.5. Thus, the sum of V_{prov} equals the number of MBBS doctors in the CHCdistrict. The weights used in vignettes analysis, denoted by W_{vig}, is given by

$$W_{vig} = W_{PSU} \times V_{prov}$$

In the analysis of district-level observations, each observation was assigned the following weight:

$$W_{PSU} = \frac{SCR\ rural\ population}{Number\ of\ districts\ in\ the\ SCR}$$

Appendix C: Additional Results

Socioeconomic status (SES) measure

As part of the Participatory Resource Assessments used to enumerate and identify health care providers in each village, information was collected on the asset ownership and status of households in each village and district to create a measure of socioeconomic status (SES) across the sample. The underlying components for this measure are reported in **Appendix Table A2**. They varied across both states and districts such that representative measures could be constructed at each level and used as predictors or covariates of quality and availability. Principal components analysis (PCA) was used at the household level to create an index of availability of these assets and statuses (membership in a scheduled caste/tribes; adult primary education completion). PCA was chosen in order to account for covariation among the index components and appropriately weight components according to their contribution to variation.

Cost and quality calculations

In our simulations of the effect of specific policy proposals on the cost and quality of health care in average rural Indian villages, we compared specific simulated outcomes to the status quo. The status quo results are reported in detail in **Appendix Table A3**. There, we report the public sector share of patient visits, the average public MBBS salary cost, the overall average cost per patient in that state, and the competence of the provider seen by the average patient in that state. The estimated share of patients visiting public sector providers in each state is calculated as the total number of (self-reported) patients seen by public sector providers divided by the total number of (self-reported) patient visits across all providers observed in all villages sampled in each state. The average public sector MBBS salary is the average salary reported by MBBS providers in each state. (No public sector providers were observed in Jharkand and Uttarakhand and therefore these values are not available.) The total cost per patient (in Indian Rupees) is the patient-share-weighted average of (a) the public sector average cost, calculated as the total salary cost divided by the total number of patients seen; and (b) the private sector average cost, calculated as the patient-weighted self-reported price per patient across private sector providers, or their total reported income divided by the number of patients, whichever is greater. The average provider competence is the patient-weighted average of the vignettes competence index across all providers in the state.

Average village availability of paramedical staff

To supplement our report of the availability of health care providers in the main text, we report the estimated availability of paramedical staff (including nurses, midwives, compounders and assistants) in each village. **Appendix Figure A1** illustrates this availability measure. The majority of these staff are public-sector staff in most states with the exception of Bihar, and the majority do not hold formal medical qualifications of any type. Since many are public-sector staff, however, they will be concentrated in a few villages and an average of, for example, 1 per village (as in Maharashtra) does not imply that every village has access to such a provider. Additionally, they are often co-located with health care providers in larger facilities. Among all such providers, a small minority are AYUSH practitioners, and only a handful in the entire national sample are MBBS-qualified.

As with our estimates of health care provider availability across states, there is no apparent correlation between health outcomes at the state level such as the under-5 mortality rate (reported here) and the number of paramedical staff available in the average village.

Provider competence score construction

We utilize the set of vignettes in which providers successfully completed some checklist questions to assess the degree to which demonstrated knowledge of diagnostic technique (as reflected in the calculated competence score) predicts demonstrated knowledge of appropriate management technique for the same vignette cases. The distribution is constructed such that the average provider in the distribution has a score of zero, and the overall distribution has a standard deviation of 1. In **Appendix Figure A2**, we illustrate the correlations between this score – estimated only from the diagnostic questions and examinations performed by each provider – with the subsequent care decisions they took in the vignettes, including the use of unnecessary antibiotics and their provision of the correct treatment for each of the cases. Across the range of competence scores, the likelihood of correctly managing the case increases from 25% to 100% across the entire range of competence scores. The average provider (score 0) demonstrated knowledge of correct management for 75% of cases and a 1-standard-deviation increase in the competence score corresponds to a change of 10 percentage points likelihood of correct treatment for any case. By contrast, the use of any medication in the vignette and the use of antibiotics specifically were very weakly correlated with the diagnostic score: providers said they would give some medication in 75% of cases and said they would give antibiotics in 25%, at all levels of diagnostic competence and treatment ability. (Note that the mean provider in this sample is somewhat better than the mean provider in the village-availability sample due to the inclusion of the broader PHC sample.)

Patterns in primary care-seeking

As part of the household survey, residents were asked if they had sought primary medical care in the past 30 days. Of the 10% of respondents who did, 66% reported that they sought care in the private sector and 33% reported they sought care from the public sector. About two-thirds reported seeking care in a village; and a third went to a town or city for care. There was no appreciable variation across sector; however, there was significant variation across states, as illustrated in **Appendix Figure A3**.

Validation of provider caseload and time use self-reporting

In a previous study involving 315 rural health care providers in West Bengal, provider surveys were combined with a participant observation (PO) exercise. In the PO exercise, a trained enumerator spent a whole day in each clinic to capture information about the actual patients and practice of each provider; this data produced matched data on 271 health care providers. This figure uses that data to compare the caseload and time-per-patient measures as self-reported in the provider survey against the caseload observed in a single day of complete clinic observation. The dashed line reports the relationship if the two were perfectly correlated; the solid line reports the relationship as observed in the data. We observe that the observed caseload is correlated with, but substantially below, the self-reported caseload in most instances, and that there is substantial heaping of self-reporting at 10, 15, and 20 patients per day, and at 10, 15, and 20 minutes per patient, as illustrated in **Appendix Figure**

A4 and A5. **Appendix Figure A6** reports the similar heaping in time-per-patient as reported in this study's provider survey.

Relationships between district SES, MBBS qualifications, and provider competence

Returning to **Table 3**, we note that the association between district SES and the knowledge of private providers disappears when we include the competence of public providers in Column 4; and we note that the MBBS coefficient is substantially reduced for private providers when we include state fixed effects. We provide some additional analysis in **Appendix Figure A7, A8, and A9** by investigating specific relationships between quality and district SES and MBBS qualifications across the various specifications.

First, we note that the competence of public providers used as a control in the regression is strongly correlated with district SES across the whole sample. However, at the state level, the relationships are significantly weaker and more varied (**Appendix Figure A8**). For MBBS qualifications, we simultaneously find that in the village sample, the states with a higher proportion of MBBS providers in the sample performed better, driving the overall relationship (but preventing significant results once state fixed-effects are used due to the small number of states with significant MBBS samples). Using the larger PHC/CHC sample, we estimate the MBBS differences at the state level, and generally find significant effects, (**Appendix Table A5** and **Appendix Figure A9**) although we note that these are primarily public sector providers and not necessarily those in the same rural areas, so we remain to have little to say about the skills of the private MBBS providers who have chosen to locate in the areas covered by the village sample.

Attrition in vignettes scoring

Since a significant proportion of the village sample was lost to follow-up in the vignettes exercise, we perform an analysis of the bias in our estimates due to this loss. First, in **Appendix Table A6**, we assess the differences between the samples with completed vignettes and those without using the same characteristics as in our demographic analysis. We observe that, overall, those with no advanced education, no other occupation, and busier and more expensive clinics are over-represented in the vignettes sample; this suggests a potential upward bias in our overall estimates of quality.

To obtain a better picture of the potential size of this bias, we conduct a two-step estimation procedure. First, using a large number of covariates, including all those above and additional demographic information about caste/tribe, religion, facility ownership, and place of birth, we use the elastic net regression procedure to machine-select those covariates that best predict completion of the vignettes module. Then, we regress both the vignettes completion and the vignettes performance on that set of covariates, reporting the results in the accompanying figure. The units for completion are percentage points; the units for performance are standard deviations. To expect substantial bias, we would need to identify some set of covariates that have large effects on both of these margins. Very few fit this criterion – full-time providers are one (leading to the potential upward biases mentioned above). The remainder of the correlates are either not selected or not significant in one or both of the selected regression models. The selected correlates and their estimated correlations with both vignettes completion and vignettes performance are reported in **Appendix Figure A10**.

Finally, using this set of correlates, we re-estimate our cost and quality results from **Figure 6**, using inverse probability weighting on the predicted likelihood of vignette completion from the elastic net model to reweight each provider's contribution to the estimates. We report both the original estimate and the re-weighted estimates in **Appendix Table A7**. We note that the changes in the estimates are both small and variable in direction, suggesting no large, predictable biases based on the initial survey data.

Table A1. Sampling and surveying of village health care providers by state

	(1) Total Providers	(2) Surveyed	(3) Not Surveyed	(4) Permanently Closed	(5) Temporarily Away	(6) Refused	(7) No Reason
**Andhra Pradesh**	131	128	3	0	3	0	0
**Assam**	40	24	16	0	11	1	4
**Bihar**	322	259	63	2	53	5	1
**Chhattisgarh**	438	408	30	10	16	4	0
**Gujarat**	115	93	22	1	15	6	0
**Haryana**	412	270	142	7	120	15	0
**Himachal Pradesh**	74	63	11	0	9	2	0
**Jharkhand**	180	140	40	1	37	2	0
**Karnataka**	103	89	14	5	8	1	0
**Kerala**	243	169	74	9	35	29	0
**Madhya Pradesh**	218	165	53	7	39	4	3
**Maharashtra**	110	101	9	0	8	0	1
**Odisha**	110	91	19	1	14	4	0
**Punjab**	329	234	95	4	54	34	3
**Rajasthan**	253	188	65	15	49	1	0
**Tamil Nadu**	53	38	15	0	5	5	5
**Uttar Pradesh**	365	288	77	10	56	7	4
**Uttarakhand**	145	121	24	0	18	6	0
**West Bengal**	694	604	90	10	68	12	0
**All India**	**4335**	**3473**	**862**	**82**	**618**	**138**	**21**

Notes: This table reports the sampling and survey completion results from each state. The first column reports the total number of providers identified in the sampled villages in that state. The second reports the total number of providers successfully surveyed. The third reports survey non-completion. The remainder of the columns report recorded reasons for survey non-completion: permanent closure, temporary unavailability, refusal, and no reason recorded. *Data source:* village provider census and survey.

Table A2. Socioeconomic status index components by state

	(1) Concrete or metal roof	(2) Piped water	(3) Electric fan	(4) Pressure cooker	(5) Mobile phone	(6) Television	(7) Bicycle	(8) Not Scheduled Caste/Tribe	(9) Adult primary education
**Andhra Pradesh**	69.6%	57.2%	89.0%	23.7%	60.2%	63.9%	10.8%	57.4%	42.2%
**Assam**	80.1%	7.7%	36.8%	22.7%	49.8%	28.7%	8.1%	77.3%	57.9%
**Bihar**	47.8%	4.1%	19.9%	15.9%	53.6%	11.7%	8.6%	77.0%	47.2%
**Chhattisgarh**	21.6%	6.7%	77.1%	18.2%	32.9%	54.1%	15.8%	60.9%	45.7%
**Gujarat**	36.4%	59.2%	82.4%	26.5%	38.5%	37.8%	20.3%	63.6%	48.5%
**Haryana**	82.7%	58.4%	86.1%	52.4%	68.3%	52.3%	26.3%	75.5%	58.2%
**Himachal Pradesh**	67.2%	73.9%	85.8%	82.0%	77.4%	78.3%	21.8%	70.3%	67.7%
**Jharkhand**	20.5%	3.3%	21.5%	8.7%	36.8%	13.2%	6.1%	49.4%	37.9%
**Karnataka**	45.3%	67.5%	52.9%	12.2%	41.6%	53.2%	15.8%	67.3%	51.1%
**Kerala**	53.3%	18.7%	90.7%	73.7%	80.4%	87.0%	28.8%	87.4%	82.8%
**Madhya Pradesh**	30.0%	18.3%	43.6%	20.1%	38.3%	26.3%	15.5%	63.8%	43.0%
**Maharashtra**	46.3%	53.9%	80.3%	53.5%	47.4%	55.1%	20.9%	65.2%	61.2%
**Odisha**	27.1%	11.1%	42.4%	11.2%	37.4%	28.1%	11.8%	54.2%	48.8%
**Punjab**	81.9%	61.0%	98.4%	72.6%	84.4%	81.7%	39.3%	60.1%	55.8%
**Rajasthan**	44.8%	36.5%	66.3%	15.4%	60.5%	31.8%	22.6%	66.3%	38.3%
**Tamil Nadu**	41.6%	92.2%	83.1%	28.3%	58.9%	93.0%	29.3%	73.2%	56.3%
**Uttar Pradesh**	59.7%	5.3%	36.4%	30.7%	48.0%	23.3%	13.5%	73.5%	44.3%
**Uttaranchal**	68.5%	56.7%	45.4%	55.5%	51.2%	46.1%	9.8%	73.8%	55.9%
**West Bengal**	41.7%	9.6%	48.2%	13.8%	41.1%	24.1%	6.9%	63.9%	44.2%

Notes: This table reports the frequency with which each component of the socioeconomic status (SES) index was observed within each state in household surveys of sampled villages. *Data source:* household survey.

Table A3. Frequency of specific behaviors in vignettes by knowledge quintile

	(1) Lowest Quintile	(2) 2nd Quintile	(3) 3rd Quintile	(4) 4th Quintile	(5) Highest Quintile
**Average Knowledge Score**	**-1.61**	**-0.45**	**0.13**	**0.64**	**1.33**
**TB: Order Sputum AFB Test**	53.4%	65.9%	74.2%	78.1%	85.9%
**Provide correct diagnose for TB**	71.9%	80.9%	90.0%	95.0%	97.4%
**TB: Correct Management (Medication)**	66.0%	73.7%	82.7%	88.5%	90.3%
**Pre-eclampsia: Ask Swelling in Feet**	3.3%	15.4%	34.4%	60.2%	82.9%
**Pre-eclampsia: Check Edema in Feet**	1.1%	7.9%	20.4%	48.0%	76.7%
**Pre-eclampsia: Correct Diagnosis**	50.9%	64.0%	75.3%	88.0%	95.4%
**Pre-eclampsia: Correct Management (Medication)**	43.0%	55.6%	67.1%	77.2%	81.2%
**Diarrhea: Asked Frequency of Stool**	22.8%	46.9%	65.6%	71.7%	81.0%
**Diarrhea: Correct Diagnosis**	58.2%	61.3%	71.1%	84.7%	92.1%
**Diarrhea: Correct Management (ORS)**	70.7%	76.7%	85.0%	92.9%	96.2%

Notes: This table groups providers who completed the vignettes into five equally-sized quintile groups and reports basic statistics for each, including: the average knowledge score in the group; the frequency with which various vignette-specific actions were taken; the frequency with which the correct diagnosis and management decision was taken for each vignette; and the likelihood that the provider recommended antibiotics in at least one of the vignettes. *Data source:* village and PHC/CHC vignettes sample.

Table A4. Cost and quality of average patient visits by state

	(1) Monthly Patients per Public MBBS	(2) Average Monthly Public MBBS Salary	(3) Public Sector Patient Share	(4) Average Public Sector Patient Cost	(5) Average Private Non-MBBS Fee	(6) Total Cost Per Patient	(7) Average Provider Competence
**Andhra Pradesh**	1245	20272	32.3%	15	14	15	1.22
**Assam**	510	22800	22.7%	39	34	21	0.78
**Bihar**	1567	23333	12.8%	12	26	26	-1.14
**Chhattisgarh**	550	17300	14.6%	32	14	18	0.10
**Gujarat**	489	91170	29.9%	97	21	51	0.75
**Haryana**	925	44500	14.0%	61	29	35	0.24
**Himachal Pradesh**	450	23000	20.9%	58	25	34	-0.48
**Jharkhand**	–	–	–	–	35	36	-0.57
**Karnataka**	822	17677	34.7%	20	17	23	1.07
**Kerala**	1292	15919	61.6%	9	58	29	1.22
**Madhya Pradesh**	1700	25000	20.3%	20	21	21	-0.33
**Maharashtra**	1200	21000	28.2%	24	46	35	0.42
**Odisha**	429	25086	41.4%	43	32	37	0.20
**Punjab**	388	22313	12.4%	46	20	24	-0.44
**Rajasthan**	458	22725	23.2%	60	31	38	0.72
**Tamil Nadu**	1848	26931	37.2%	15	19	20	1.19
**Uttar Pradesh**	293	22111	11.1%	57	17	24	-1.09
**Uttarakhand**	–	–	–	–	12	13	-0.17
**West Bengal**	1143	29694	9.2%	28	28	29	0.02

Notes: This table reports the public sector share of patient visits, the average public MBBS salary cost, the overall average cost per patient in that state, and the competence of the provider seen by the average patient in that state. The estimated share of patients visiting public sector providers in each state is calculated as the total number of (self-reported) patients seen by public sector providers divided by the total number of (self-reported) patient visits across all providers observed in all villages sampled in each state. The average public sector MBBS salary is the average salary reported by MBBS providers in each state. (No public sector providers were observed in Jharkand and Uttarakhand and therefore these values are not available.) The total cost per patient (in Indian Rupees) is the patient-share-weighted average of (a) the public sector average cost, calculated as the total salary cost divided by the total number of patients seen; and (b) the private sector average cost, calculated as the patient-weighted self-reported price per patient across private sector providers, or their total reported income divided by the number of patients, whichever is greater. The average provider competence is the patient-weighted average of the vignettes competence index across all providers in the state. *Data source:* village provider census and survey; village vignettes sample.

Table A5. Relationship between MBBS degree and provider competence by state

	(1)	(2)	(3)	(4)	(5)	(6)
	MBBS Providers	MBBS Share	Non-MBBS Providers	Non-MBBS Share	MBBS Mean Competence	Non-MBBS Mean Competence
**Andhra Pradesh**	72	49.7%	73	50.3%	0.73	0.35
**Assam**	197	77%	59	23%	0.51	0.28
**Bihar**	152	52.6%	137	47.4%	-0.82	-2.49
**Chhattisgarh**	40	19.3%	167	80.7%	1.11	-0.22
**Gujarat**	68	43.3%	89	56.7%	1.22	0.50
**Haryana**	127	51.8%	118	48.2%	0.15	-0.66
**Himachal Pradesh**	90	71.4%	36	28.6%	0.17	-1.27
**Jharkhand**	130	61.3%	82	38.7%	-1.00	-2.28
**Karnataka**	61	49.2%	63	50.8%	0.79	0.63
**Kerala**	114	78.6%	31	21.4%	0.89	0.53
**Madhya Pradesh**	66	40.5%	97	59.5%	0.05	-1.04
**Maharashtra**	70	36.6%	121	63.4%	0.86	0.01
**Odisha**	84	38.5%	134	61.5%	0.33	-0.08
**Punjab**	75	31.4%	164	68.6%	-0.26	-1.09
**Rajasthan**	89	40.8%	129	59.2%	0.69	0.27
**Tamil Nadu**	66	73.3%	24	26.7%	1.07	0.62
**Uttar Pradesh**	64	25.2%	190	74.8%	-0.57	-2.13
**Uttarakhand**	60	40.5%	88	59.5%	-0.14	-1.44
**West Bengal**	113	28.1%	289	71.9%	-0.40	-0.52

Notes: This table reports the number and share of providers in the complete vignettes sample with MBBS degrees in each state, as well as their average estimated competence by MBBS qualification. *Data source:* village and PHC/CHC vignettes sample.

Table A6. Relationships between provider characteristics and vignette completion

	(1)	(2)	(3)	(4)	(5)
	Completed Vignettes		Did Not Complete		T-test
	N	Mean/SE	N	Mean/SE	Difference
**Panel A: All Providers**					
Male	1452	0.919	2009	0.877	0.042***
Have Grade 10 Education	1458	0.231	2015	0.323	-0.092***
Have Grade 10+2 Education	1458	0.431	2015	0.388	0.044***
Have Advanced Education	1458	0.333	2015	0.285	0.048***
Have No Other Occupation	1455	0.710	2007	0.635	0.075***
Age	1447	40.288	1988	40.469	-0.181
Caseload x10	1456	1.682	2012	1.514	0.169**
Total Fee x10	1416	2.402	1951	2.089	0.313***
Time per Patient x10	1457	1.210	2011	1.158	0.052**
MBBS	1458	0.088	2015	0.067	0.021**
AYUSH	1458	0.274	2015	0.220	0.054***
Informal	1458	0.638	2015	0.713	-0.075***
Private	1458	0.887	2015	0.866	0.021*
**Panel B: MBBS Providers**					
Male	126	0.770	135	0.741	0.029
Have Grade 10 Education	129	0.016	135	0.000	0.016
Have Grade 10+2 Education	129	0.372	135	0.393	-0.020
Have Advanced Education	129	0.612	135	0.607	0.005
Have No Other Occupation	128	0.922	135	0.970	-0.048*
Age	127	42.142	135	41.504	0.638
Caseload x10	129	4.993	135	4.655	0.338
Total Fee x10	119	1.664	130	2.193	-0.529
Time per Patient x10	129	0.981	135	0.863	0.118*
Private	129	0.504	135	0.444	0.059
**Panel C: Non-MBBS Providers**					
Male	1326	0.933	1874	0.886	0.047***
Have Grade 10 Education	1329	0.252	1880	0.346	-0.094***
Have Grade 10+2 Education	1329	0.437	1880	0.387	0.050***
Have Advanced Education	1329	0.305	1880	0.262	0.044***
Have No Other Occupation	1327	0.690	1872	0.611	0.078***
Age	1320	40.110	1853	40.393	-0.284
Caseload x10	1327	1.361	1877	1.288	0.073
Total Fee x10	1297	2.470	1821	2.082	0.388***
Time per Patient x10	1328	1.232	1876	1.179	0.053**
Private	1329	0.924	1880	0.896	0.028***

Notes: This table reports balance checks from the sampled providers in the village survey that did and did not complete the vignettes samples. *Data source:* village provider census and survey.

Table A7. Effect of IPW reweighting on cost and quality estimates

	(1) Cost per Patient (Unweighted)	(2) Cost per Patient (Reweighted)	(3) Average Quality (Unweighted)	(4) Average Quality (Reweighted)
Andhra Pradesh	14.74	15.20	1.22	1.25
Assam	21.18	24.92	0.78	0.71
Bihar	26.39	26.67	-1.14	-1.13
Chhattisgarh	18.37	20.33	0.10	0.20
Gujarat	50.56	53.14	0.75	0.72
Haryana	34.69	35.23	0.24	0.21
Himachal Pradesh	33.79	33.74	-0.48	-0.52
Jharkhand	35.69	37.49	-0.57	-0.51
Karnataka	23.33	24.35	1.07	1.09
Kerala	29.36	26.93	1.22	1.21
Madhya Pradesh	21.13	21.97	-0.33	-0.31
Maharashtra	35.20	38.19	0.42	0.44
Odisha	36.79	38.20	0.20	0.23
Punjab	23.62	23.95	-0.44	-0.47
Rajasthan	38.38	39.27	0.72	0.74
Tamil Nadu	19.65	19.78	1.19	1.16
Uttar Pradesh	23.83	24.10	-1.09	-1.11
Uttarakhand	12.98	13.08	-0.17	-0.20
West Bengal	28.86	30.18	0.02	0.02

Notes: This table reports the cost in Indian rupees per visit seen against the average caseload-weighted provider competence for each state, using both the original unweighted values as well as values calculated from weighting each provider by the inverse of the likelihood of their participation in the vignettes sample using the elastic net model. The cost per visit in the public sector is calculated as the sum of the total public sector wage bill using reported monthly salaries from all providers and dividing this total by the total number of patients seen each day multiplied by 20 working days per month. For the private sector, it is the greater of total reported medical income divided by the number of patients seen per day times 20 working days; or the self-reported fee per patient. The mean provider competence corresponds to a score of zero and the standard deviation is 1. *Data source:* village provider census and survey; village and PHC/CHC vignettes sample.

Figure A1. Average village availability of paramedical staff by state

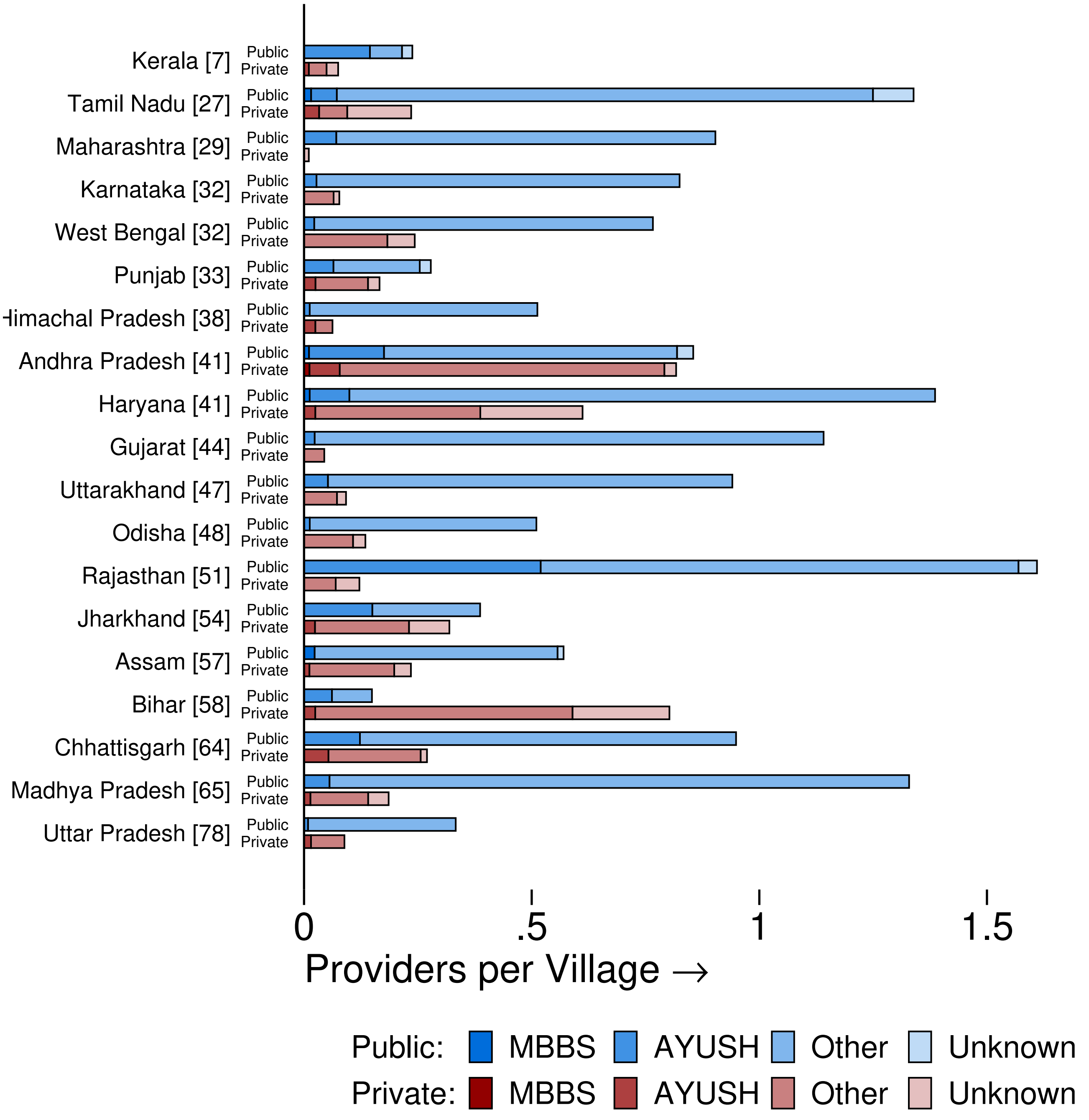

Notes: This figure reports the number of paramedical health staff of each type and sector available in the average village in each state. States are ordered by the under-five mortality rate per 1,000 live births over the five-year period covered by the National Family Health Survey (NFHS-4) for 2015-16 (reported in brackets). Health care providers are defined as surveyed health care workers who are do not fall under the definition of primary care providers at health care facilities, excluding chemists. "Paramedical" staff are therefore staff such as nurses/GNMs, ANM/VHNs, MPW/MNAs, and compounders and assistants. They are categorized by sector (public or private) and by the type of medical education reported: an MBBS degree, or the equivalent of an MD; an AYUSH degree, or a licensed practitioner of alternative or traditional medicine; other, or no recognized formal medical degree; and unknown, or no response. *Data source:* village provider census and survey.

Figure A2. Correlations between provider competence score and vignette actions

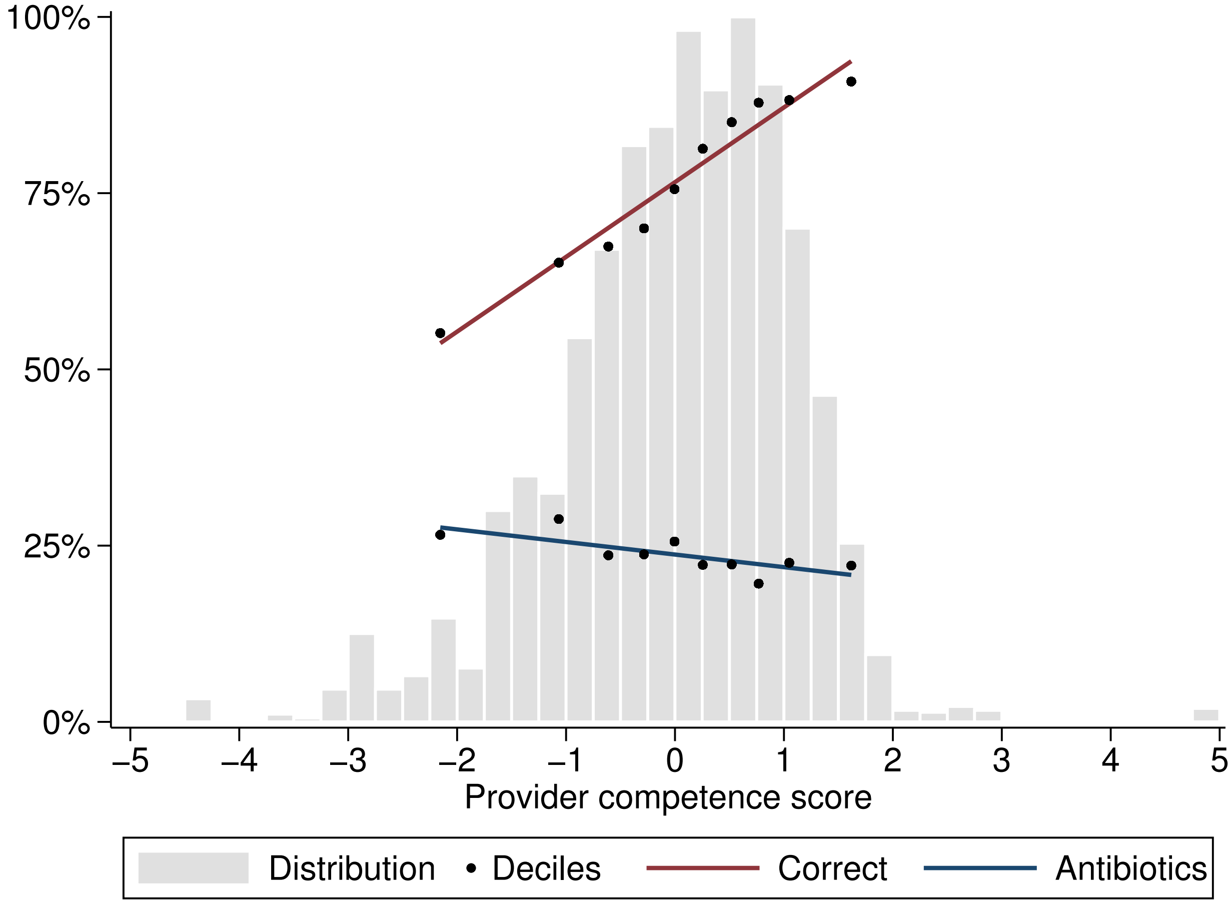

Notes: This figure reports the full-sample correlations between the estimated provider-level diagnostic competence score from all medical vignettes with specific actions in each vignette. These actions are correct management of the vignette and any (unnecessary) antibiotics prescribed in the vignette. The correlations are produced by calculating decile bins of the vignette-based competence score, and calculating the average rate of these two measures within those bins. The correlation fits are linear across these decile bins. *Data source:* village and PHC/CHC vignettes sample.

Figure A3. Share of within-village visits among private primary care seekers by state

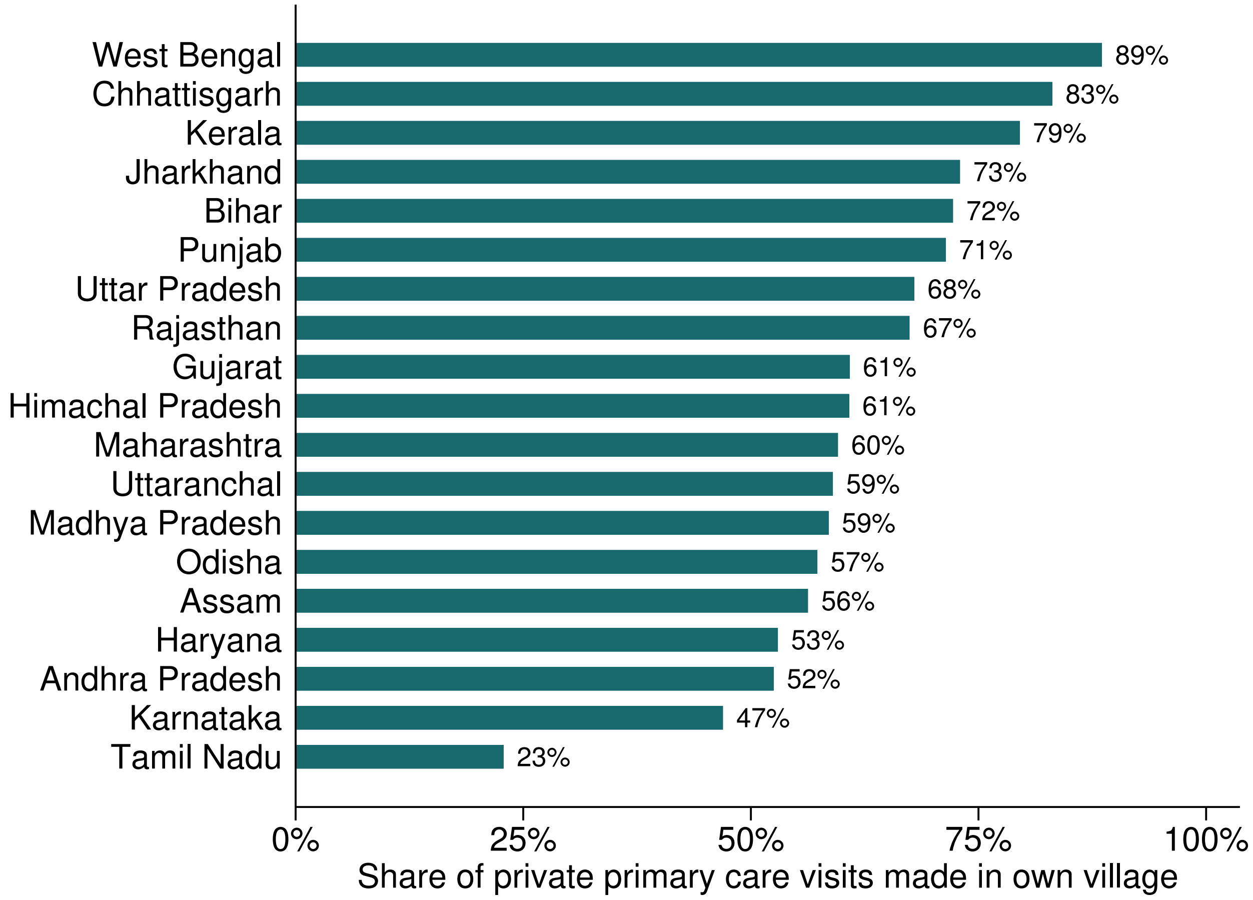

Notes: This figure reports the total share of within-village visits among private primary care seekers each state. Data is collected in household surveys, among individuals who reported seeking primary care in the private sector. *Data source:* village provider census and survey; household survey.

Figure A4. Self-reported compared with observed caseload in Birbhum study

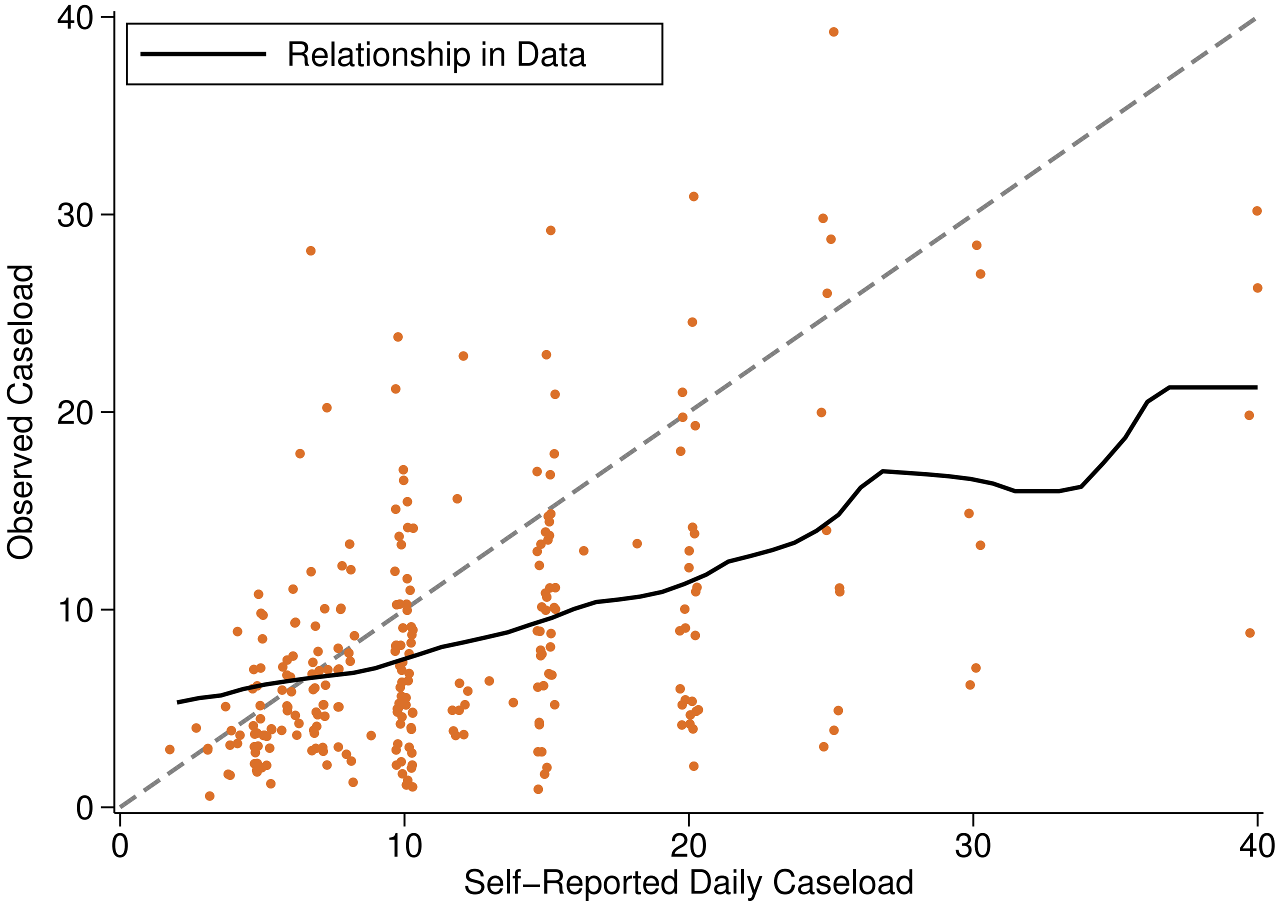

Notes: This figure uses data from a previous study to compare the caseload as self-reported in the provider survey against the caseload observed in a single day of complete clinic observation. The dashed line reports the relationship if the two were perfectly correlated; the solid line reports the relationship as observed in the data. N=271 rural health care providers in West Bengal. *Data source:* Das, Jishnu, Abhijit Chowdhury, Reshmaan Hussam, and Abhijit V. Banerjee. "The impact of training informal health care providers in India: A randomized controlled trial." *Science* 354, no. 6308 (2016): aaf7384.

Figure A5. Self-reported compared with observed time with patients in Birbhum study

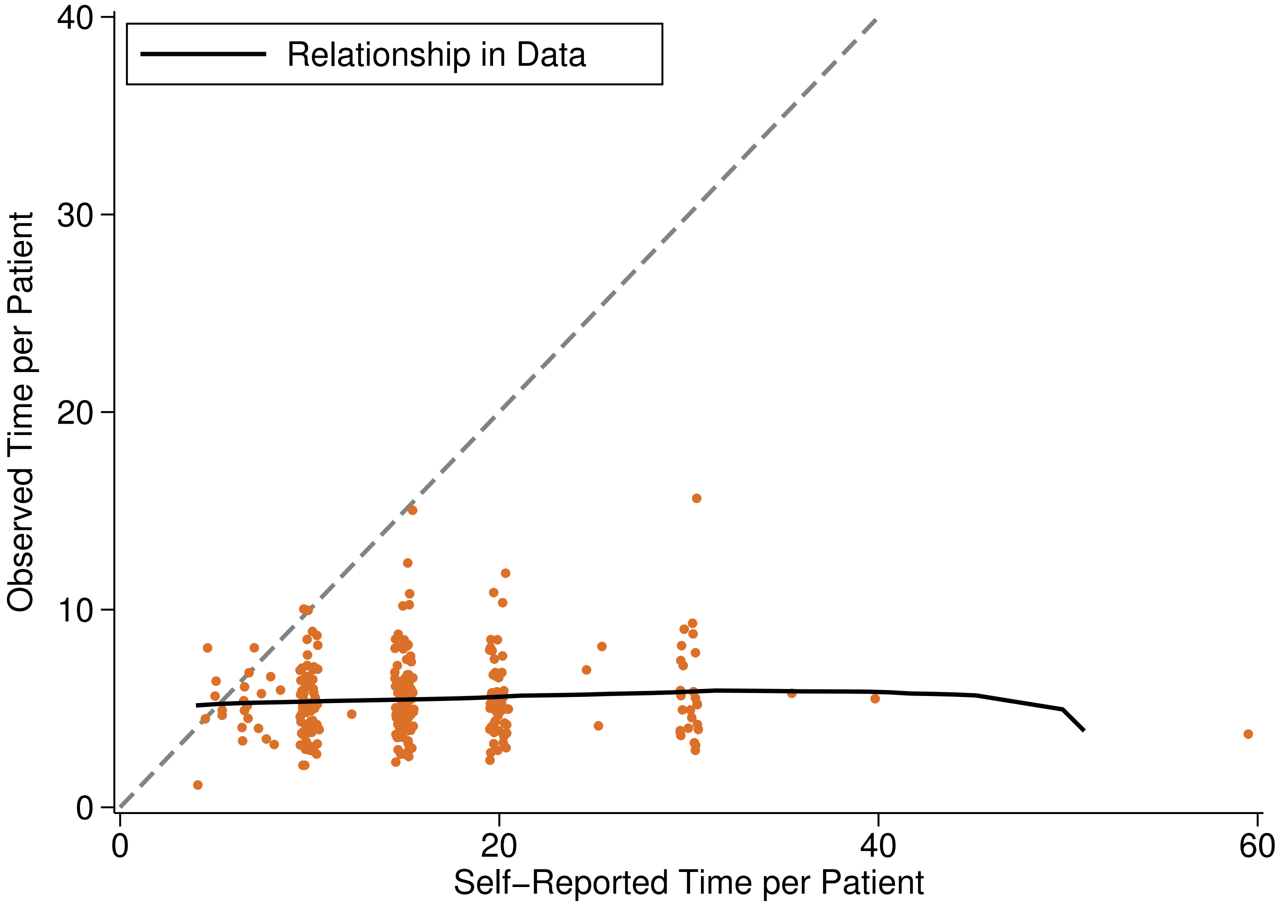

Notes: This figure uses data from a previous study to compare the time per patient as self-reported in the provider survey against the caseload observed in a single day of complete clinic observation. The dashed line reports the relationship if the two were perfectly correlated; the solid line reports the relationship as observed in the data. N=271 rural health care providers in West Bengal. *Data source:* Das, Jishnu, Abhijit Chowdhury, Reshmaan Hussam, and Abhijit V. Banerjee. "The impact of training informal health care providers in India: A randomized controlled trial." *Science* 354, no. 6308 (2016): aaf7384.

Figure A6. Data heaping of self-reported time with patients in MAQARI data

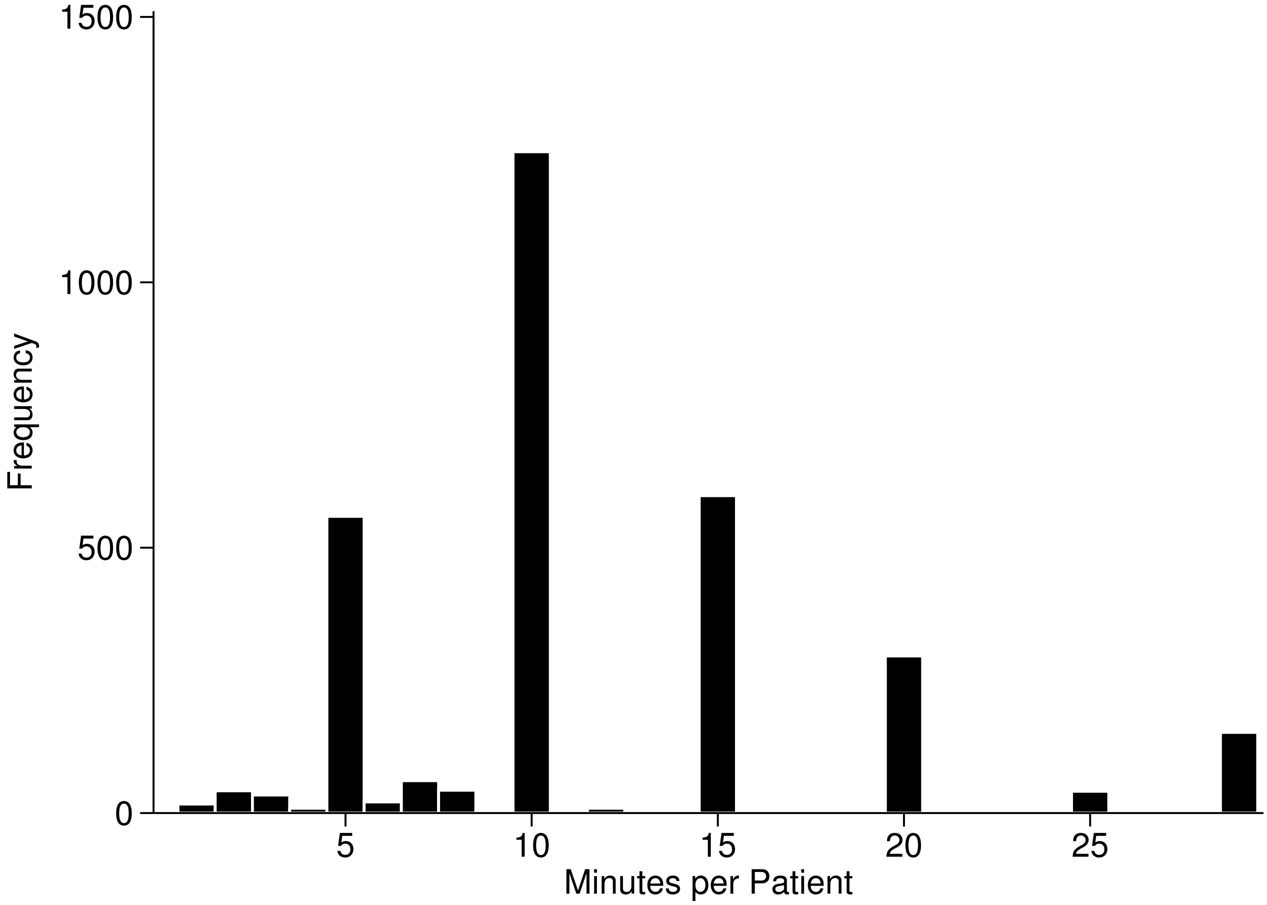

Notes: This figure illustrates the complete distribution of responses to the question asking how much time the provider spends with the average patient. The question was posed as an open-ended response. *Data source:* village provider census and survey.

Figure A7. Relationship between district SES and public provider competence

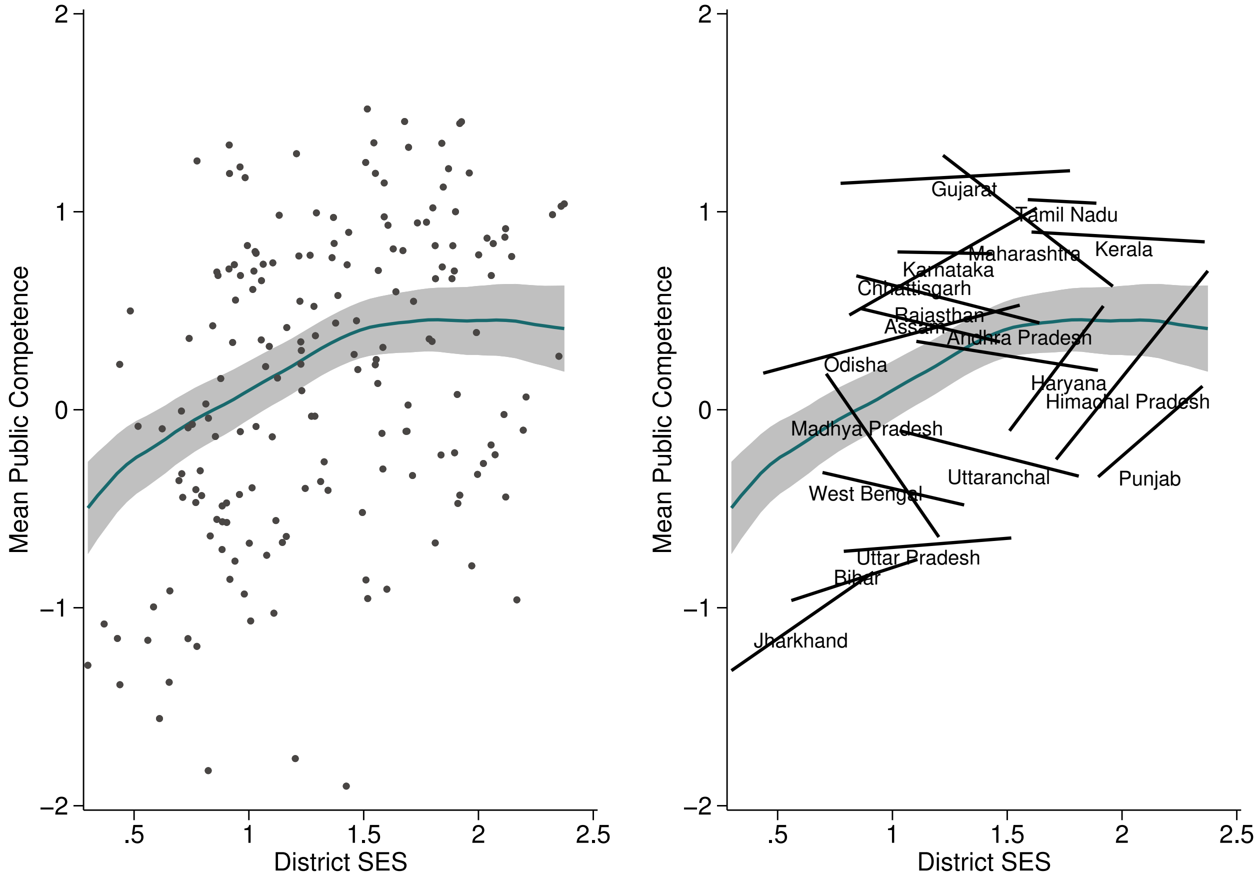

Notes: This figure illustrates the relationships between district socioeconomic status and the average competence of public sector providers as recorded in the clinical vignettes at the district level. In the first panel, all districts are reported and the overall correlation is graphed using a nonparametric LOWESS fit. In the second panel, the same overall correlation is displayed, and the individual linear correlations for each state are graphed over their supports. N=2,359 providers in 188 districts. *Data source:* household survey and PHC/CHC vignettes sample.

Figure A8. Relationship between state MBBS share and provider competence

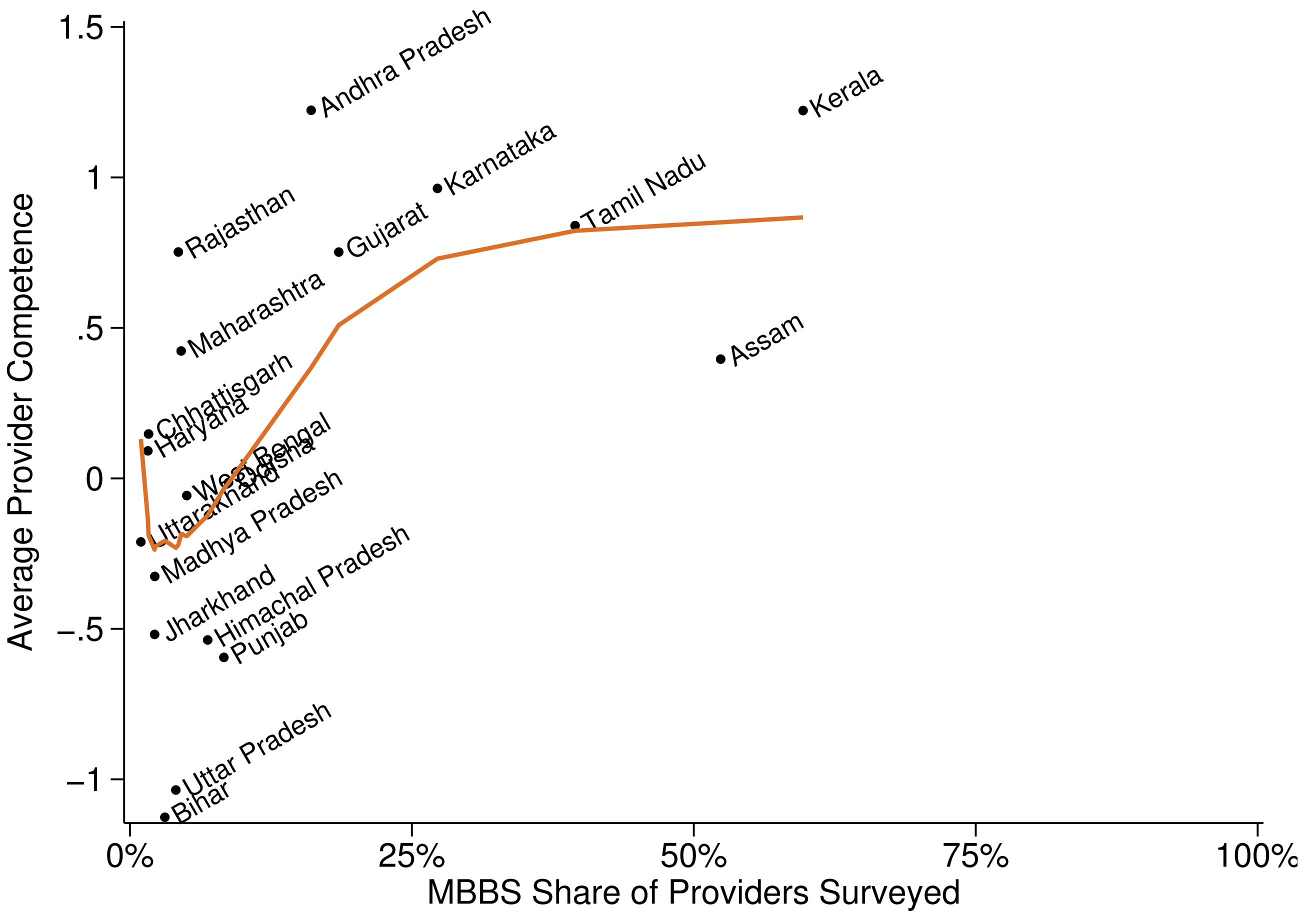

Notes: This figure illustrates the relationship between the share of MBBS providers in the village sample. *Data source:* village provider survey and vignettes sample.

Figure A9: Relationship between MBBS degree and provider competence by state

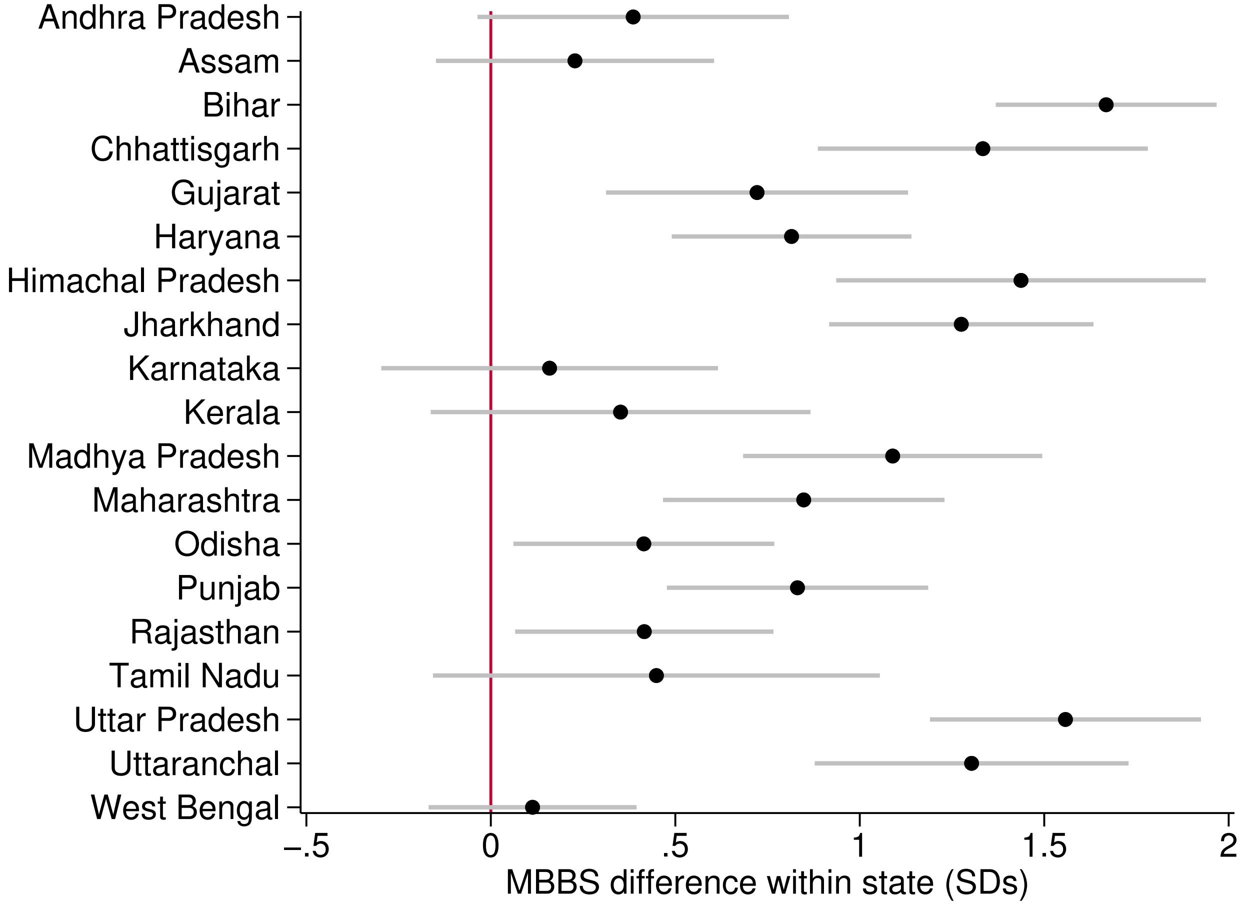

Notes: This figure illustrates the average differences between the estimated competence of MBBS and non-MBBS providers in the complete vignettes data. The estimated differences and standard errors are obtained from a regression which fully interacts MBBS qualification with state. N=2,359 providers. *Data source:* village survey and village and PHC/CHC vignettes samples.

Figure A10. Relationship between selection characteristics and vignettes results

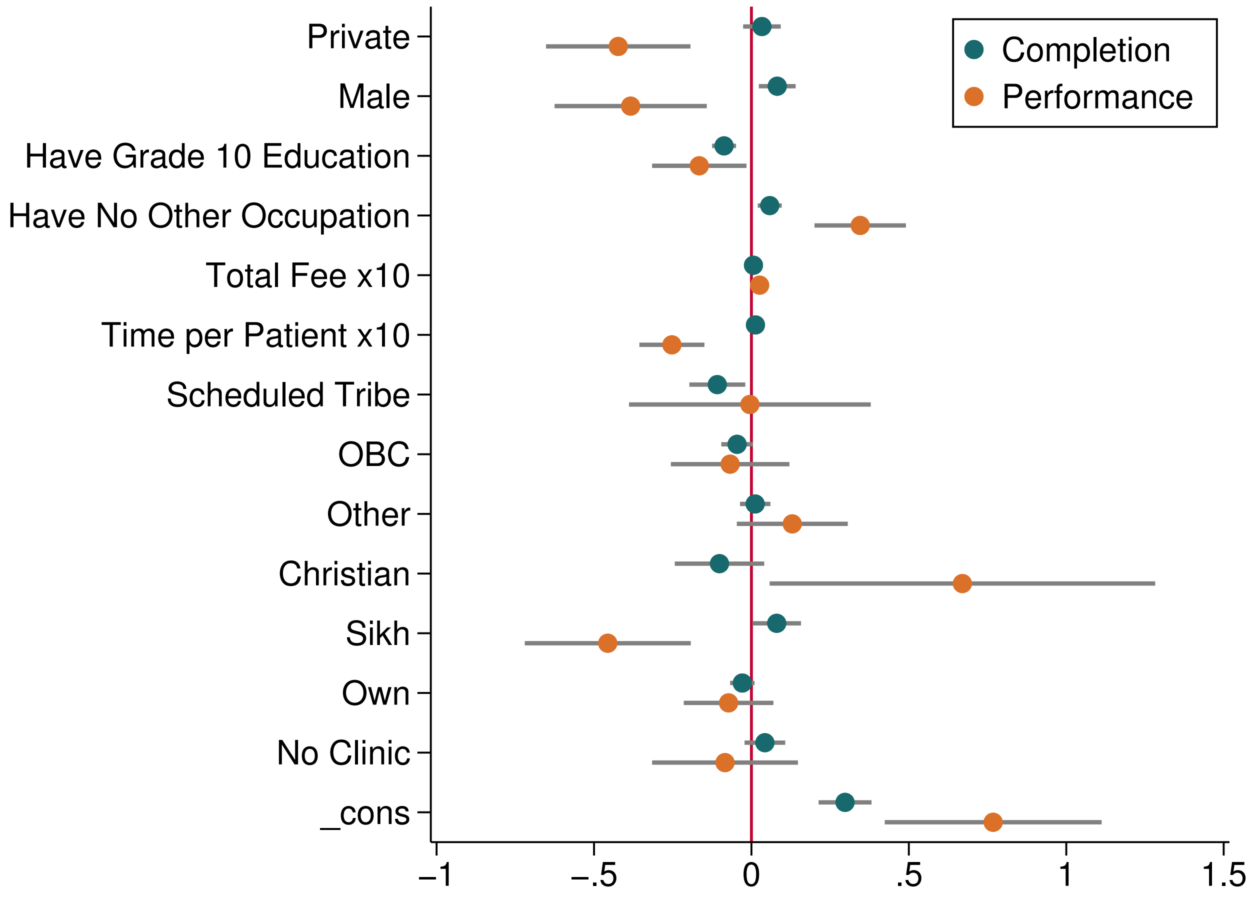

Notes: This figure illustrates the estimated relationships between machine-selected covariates and completion and performance in the vignettes sample for providers observed in the village survey round. First using our original demographic characteristics and additional demographic information about caste/tribe, religion, facility ownership, and place of birth, we use the elastic net regression procedure to machine-select those covariates that best predict completion of the vignettes module. We then regress both the vignettes completion and the vignettes performance on that set of covariates, reporting the estimated coefficients in this figure. Units for vignettes completion are percentage points (a coefficient of +0.5 represents a 50% higher likelihood of completing the vignette); units for vignettes performance are standard errors relative to the mean performance. N=3,338 providers (vignette completion) and 1,406 providers (vignette performance). *Data source:* village survey and vignettes sample.
